# A Cre Driver Line for Genetic Targeting of Kappa Opioid Receptor Expressing Cells

**DOI:** 10.1523/ENEURO.0043-23.2023

**Published:** 2023-07-11

**Authors:** Franciely Paliarin, Chelsea Duplantis, Andrea F. Jones, Jessica Cucinello-Ragland, Samhita Basavanhalli, Emily Blaze, Evan Doré, Anna Isabella Neel, Haiguo Sun, Rong Chen, Scott Edwards, Nicholas W. Gilpin, Robert O. Messing, Rajani Maiya

**Affiliations:** 1 Department of Physiology, LSU Health Sciences Center, New Orleans, Louisiana 70112; 2 Department of Physiology and Pharmacology, Wake Forest University School of Medicine, Winston-Salem, North Carolina 27157; 3 Department of Neuroscience and Waggoner Center for Alcohol and Addiction Research, The University of Texas at Austin, Austin, Texas 78712

**Keywords:** anxiety, conditioned place aversion, dynorphin, genetic access, knock-in mice, social interaction

## Abstract

Here we describe the generation and characterization of a *Cre* knock-in mouse line that harbors a Cre insertion in the 3′UTR of the κ opioid receptor gene (*Oprk1*) locus and provides genetic access to populations of κ opioid receptor (KOR)-expressing neurons throughout the brain. Using a combination of techniques including RNA *in situ* hybridization and immunohistochemistry, we report that Cre is expressed with high fidelity in KOR-expressing cells throughout the brain in this mouse line. We also provide evidence that Cre insertion does not alter basal KOR function. Baseline anxiety-like behaviors and nociceptive thresholds are unaltered in *Oprk1-Cre* mice. Chemogenetic activation of KOR-expressing cells in the basolateral amygdala (BLA^KOR^ cells) resulted in several sex-specific effects on anxiety-like and aversive behaviors. Activation led to decreased anxiety-like behavior on the elevated plus maze and increased sociability in female but not in male *Oprk1-Cre* mice. Activation of BLA^KOR^ cells also attenuated KOR agonist-induced conditioned place aversion (CPA) in male *Oprk1-Cre* mice. Overall, these results suggest a potential role for BLA^KOR^ cells in regulating anxiety-like behaviors and KOR-agonist mediated CPA. In summary, these results provide evidence for the utility of the newly generated *Oprk1-Cre* mice in assessing localization, anatomy, and function of KOR circuits throughout the brain.

## Significance Statement

Here we report the generation and characterization of an *Oprk1-Cre* mouse line that harbors Cre insertion in the 3′UTR of the *Oprk1* locus. There is high fidelity of Cre expression to KOR-expressing cells throughout the brain in this mouse line and Cre insertion does not impair KOR function. Chemogenetic activation of BLA^KORs^ led to sex-specific effects on anxiety-like behaviors and attenuated KOR agonist-induced conditioned place aversion. These results provide evidence for the utility of the newly generated *Oprk1-Cre* mice to interrogate KOR function in discreet circuits.

## Introduction

The κ opioid receptor (KOR) is a member of the opioid receptor family that is widely expressed throughout the CNS and peripheral nervous system. The KORs belong to the seven-transmembrane G-protein-coupled receptor superfamily and are coupled to Gα_i/o_ proteins. Dynorphin (Dyn) is the primary endogenous ligand for KORs. Upon agonist binding to KORs, activated Gα_i/o_ subunits inhibit adenylyl cyclase and therefore decreased cAMP production. The released G_βγ_ subunits block Ca^+2^ channels and activate GIRK channels (Al-Hasani and Bruchas, 2011; Crowley and Kash, 2015). The net result of these events is hyperpolarization of the cell and therefore decreased likelihood of action potential firing. In addition, agonist binding at KORs activates other intracellular signaling cascades including the extracellular signal-regulated kinase (ERK) 1/2, p38 MAPK, and JNK pathways (Bruchas et al., 2006, 2007a, b). A large body of preclinical literature implicates a role for the Dyn/KOR system in regulating mood, reward-related processes, pain, and cognition (Bruchas et al., 2010; Al-Hasani and Bruchas, 2011; Crowley and Kash, 2015; Abraham et al., 2021). Activation of the KORs *in vivo* results in dysphoric and anhedonic states and induces conditioned place avoidance (CPA) in rodents (Bruchas et al., 2010; Al-Hasani and Bruchas, 2011; Crowley and Kash, 2015; Abraham et al., 2021). However, insight into the role of KORs in these behaviors was primarily derived from pharmacological and genetic manipulation studies leaving open questions regarding the precise cellular and circuit location of KORs mediating these behavioral effects.

KORs and their endogenous ligand Dyn are widely expressed in the adult brain, including basolateral amygdala (BLA) nucleus and central nucleus of the amygdala (CeA), the nucleus accumbens (NAc), caudate, putamen, paraventricular thalamus (PVT), hypothalamus, and ventral tegmental area (VTA; DePaoli et al., 1994). The Dyn/KOR system is heavily implicated in mediating the behavioral effects of stress (Bruchas et al., 2010; Crowley and Kash, 2015). Stressful events can cause the release of Dyn in a variety of brain regions, and Dyn-mediated activation of KORs is thought to contribute to dysphoria and negative affective states that result from chronic stress (Bruchas et al., 2010; Crowley and Kash, 2015). Moreover, there is also strong evidence that KORs play a role in substance use disorders (Nestler, 1996). Specifically, the Dyn/KOR system is thought to contribute to the negative affective and dysphoric state referred to as “the dark side” of addiction that results from chronic drug use and leads to further escalation of drug intake (Chavkin and Koob, 2016). Stress is a risk factor for major depressive disorders (Nemeroff and Vale, 2005) and KOR antagonists are currently being investigated for therapeutic efficacy in the treatment of depression (Krystal et al., 2020). Further, KORs are also expressed in nociceptor populations in the periphery as well as in brain regions involved in pain. KOR agonists have marked analgesic properties and are also being investigated as a therapeutic target for acute and chronic pain (Snyder et al., 2018). KOR agonists are also antipruritic and inhibit itching caused by a variety of pruritogens (Nguyen et al., 2021; Inan and Cowan, 2022). Hence, KORs have emerged as promising therapeutic candidates for the treatment of a wide range of neuropsychiatric disorders ranging from depression and addiction to pain and pruritis.

Despite tremendous interest in therapeutics targeting KORs, the cellular and circuit mechanisms by which KORs mediate these diverse behaviors remain largely unknown. Tools to manipulate KOR-expressing cells in the brain and periphery were lacking until recently. A recently generated *Oprk1-Cre* mouse harbors Cre insertion in the second exon of the *Oprk1* locus that may disrupt endogenous gene transcription (Cai et al., 2016). In this article, we describe the generation and characterization of a novel *Oprk1-Cre* mouse that harbors a Cre insertion in the 3′UTR of the *Oprk1* locus. We verify the fidelity of Cre expression in this mouse line using a variety of methods. We also show that Cre insertion into the *Oprk1* locus does not alter KOR function or baseline anxiety-like and pain-like behaviors. We also show that the activation of BLA^KORs^ is anxiolytic in females and attenuates KOR agonist-induced CPA in males.

## Materials and Methods

### Mice

*Oprk1-Cre* mice harboring a *Cre* insertion in the 3′UTR of the *Oprk1* locus were generated by Cyagen using homologous recombination. Briefly, the TGA stop codon at the *Oprk1* locus was replaced by a “*P2A-Cre*” cassette (Extended Data Fig. 1-1). The targeting vector contained homology arms that were generated using the BAC (bacterial artificial chromosome) clone as a template. The Neo cassette in the targeting vector was flanked by self-deletion anchor sites. C57BL/6N embryonic stem (ES) cells were used for gene targeting. A targeted ES cell clone harboring the correct insertion was injected into C57BL/6N embryos before being implanted into a pseudopregnant CD1 female. Founder animals were identified by coat color, and their germline transmission was confirmed by breeding with C57BL/6N females and subsequent genotyping of the offspring. Mice were genotyped by Transnetyx. All mice were provided access to food and water *ad libitum* and housed in reverse light/dark cycle (on at 10:00 A.M., off 10:00 P.M.). Animals that went surgical procedures were singly housed postsurgery before behavioral analyses. *Oprk1-Cre* mice that were backcrossed to C57BL/6J mice for at least three generations were used in this study. Only heterozygous *Oprk1-Cre* mice and their wild-type (WT) littermates were used for all behavioral, Western blotting, immunohistochemistry (IHC), [^35^S] GTPγS, and quantitative PCR (qPCR) experiments. Homozygous Cre mice were used for *in situ* hybridization to verify the fidelity of *Cre* and *Oprk1* expression. For behavioral experiments, male and female mice were tested concurrently in mixed sex cohorts. Floxed L10A-EGFP mice were obtained from Bradford Lowell (Harvard University, Boston, MA) and have been previously described (Krashes et al., 2014).

**Figure 1.**
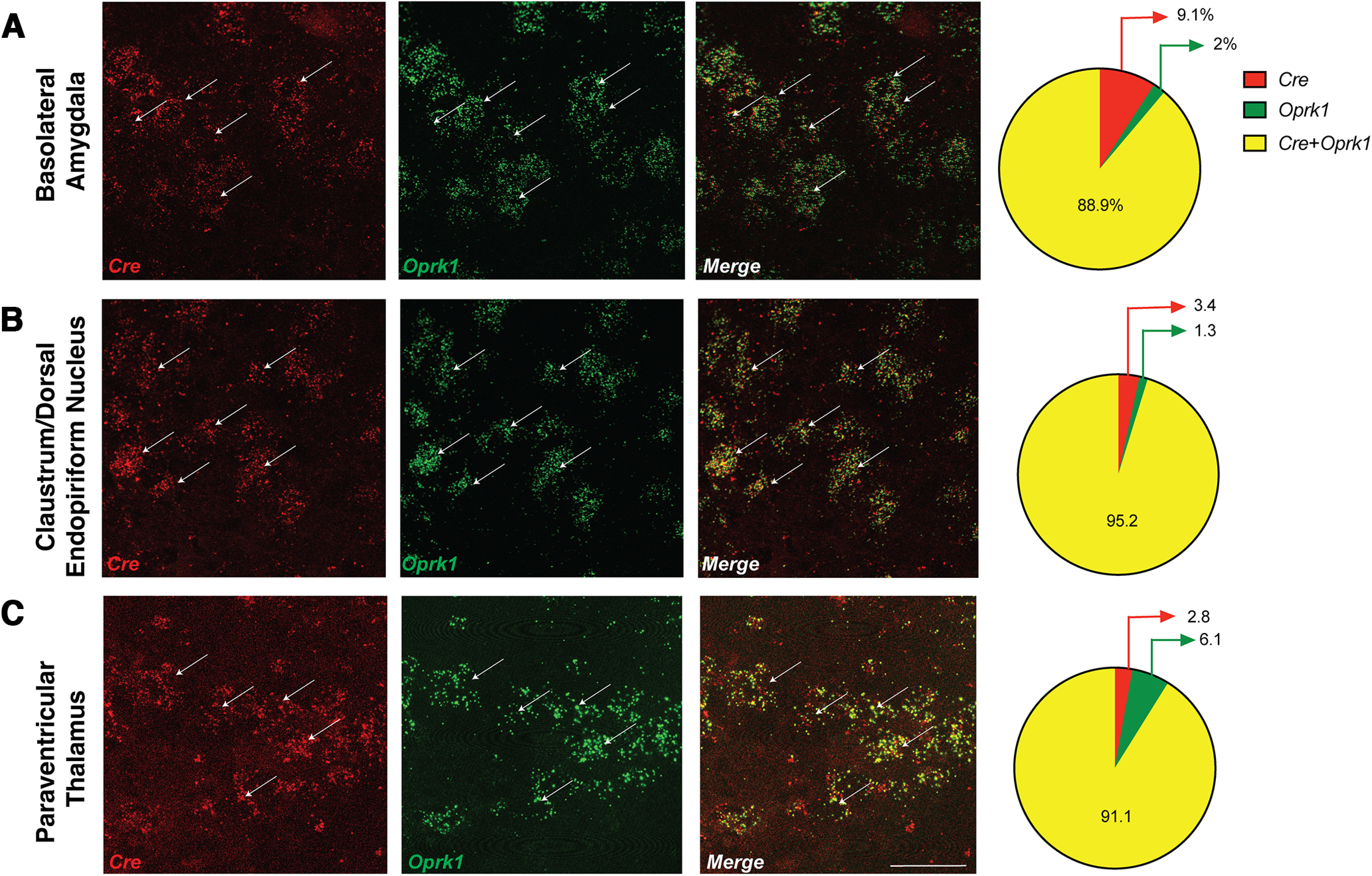
Colocalization of Oprk1 and Cre. ***A***–***C***, *In situ* hybridization using probes targeting *Cre* recombinase and mouse *Oprk1* reveal 80–90% colocalization between *Oprk1* and *Cre* transcripts across the following three brain regions: the BLA (***A***), claustrum/dorsal endopiriform nucleus (***B***), and PVT (***C***). *N* = 2 male mice; *N* = 1 female mouse. Scale bar, 50 µm. See Extended Data Figure 1-1 for generation of *Oprk1-Cre* transgenic mouse.

10.1523/ENEURO.0043-23.2023.f1-1 Figure 1-1 Schematic illustrating insertion of Cre recombinase into the 3′UTR of the KOR locus. Download Figure 1-1, TIF file.

### Drugs and viral vectors

U-50488 (Tocris Bioscience) was dissolved in saline and injected intraperitoneally at 10 mg/kg for biochemistry experiments and 2.5 mg/kg for CPA experiments (10 ml/kg). Clozapine *N*-oxide (CNO; free base) was obtained from Hello Bio and dissolved in saline containing 0.5% DMSO. Mice were injected with 3–5 mg/kg CNO (at 10 ml/kg) 30 min before behavioral testing. The Cre-dependent viral vectors AAV8-hSyn-DIO-hM3DQ-mCherry, AAV8-hSyn-DIO-mCherry, AAV8-hSyn-DIO-EGFP were obtained from Addgene. The titers of all AAVs were ∼3–5 × 10^12^ infectious units/ml.

### Stereotaxic surgeries

Adeno-associated virus (AAV; 100 nl) encoding either Cre-dependent mCherry or hM3DQ, as described previously (Lasek and Azouaou, 2010; Maiya et al., 2021), were infused bilaterally into the BLA of male and female *Oprk1-Cre* mice using an injector (Nanoject III, Drummond Scientific). The coordinates for BLA were as follows: anteroposterior, −1.6 mm from skull; mediolateral, ±3.25 mm from skull; dorsoventral, −4.5 mm from skull. Mice were allowed to recover for 2 weeks before behavioral testing.

### Immunohistochemistry for Cre recombinase

Mice were perfused transcardially with PBS followed by 4% paraformaldehyde (PFA) in PBS, pH 7.4. Brains were extracted, postfixed overnight in the same fixative, and cryoprotected in 30% sucrose in PBS at 4°C. Brains were sectioned at 40 μm thickness on a cryostat, and free-floating sections were collected in PBS. Sections were washed three times in PBS containing 0.2% Triton X-100 (PBST) for 10 min at 27°C, incubated in 3% hydrogen peroxide for 5 min, and washed 3 × 10 min in PBS. Sections were then blocked for 1 h in PBS containing 0.3% Triton X-100 and 1% BSA at 27°C, and incubated in 1:700 dilution of rabbit anti-Cre antibody (gift from C. Kellendonk, Columbia University, New York, NY) for 24 h at 4°C on a rocker. Sections were then washed 1× in 0.1 m Tris-Cl, pH 7.4, 150 mm NaCl, and 0.3% Triton X-100 (TNT buffer) for 10 min followed by incubation in 0.1 m Tris-Cl, pH 7.4, 150 mm NaCl, and 0.5% TSA blocking reagent (TNB buffer; Akoya Biosciences) for 30 min. This was followed by incubation in ImmPRESS HRP Horse Anti-Rabbit HRP-conjugated secondary antibody (Vector Laboratories) for 1 h. Sections were then washed 4× for 5 min in TNT buffer followed by incubation in trichostatin A (TSA) reagent (fluorescein in 1:50 TSA amplification diluent; Akoya Biosciences). Following this incubation, sections were washed 3× for 10 min in TNT buffer, mounted on slides and coverslipped with Fluormount G (Southern Biotech) mounting media containing DAPI. Tiled images of the entire brain were acquired at 20× resolution on a microscope (model BX51, Olympus).

### RNA *in situ* hybridization

We performed fluorescence *in situ* hybridization to examine colocalization between *Oprk1* and *Cre* transcripts. Mouse brains were rapidly extracted by decapitation and flash frozen in a dry ice/isopentane bath. Coronal sections (12 µm) were obtained and mounted on a glass slide. *In situ* hybridization was performed using RNAScope Fluorescent Multiplex Kit (Advanced Cell Diagnostics). The following probes were used for RNAScope: *Oprk1* (catalog #31611); *Cre* (catalog #423321); and EGFP-04 (catalog #538851). Slides were coverslipped with Fluormount-G with DAPI (Southern Biotech) and imaged using a confocal microscope (Leica) at 20× resolution. The extent of colocalization between *Oprk1* and *Cre* was determined using Fiji as previously described (Schindelin et al., 2012; Pomrenze et al., 2019; Maiya et al., 2021). To determine colocalization between virally expressed Cre-dependent *EGFP* and *Oprk1*, mice were killed 2 weeks postsurgery.

### Validation of designer receptors activated by designer drug-induced activation of BLA^KOR^ neurons

*Oprk1-Cre* mice expressing hM3DQ in the BLA were administered either vehicle or CNO (3 mg/kg) 90 min before being killed. Mice were perfused transcardially with PFA, and the brain was removed, postfixed, and processed for IHC, as described in subsection Immunohistochemistry for Cre recombinase. Sections were incubated with Rabbit c-Fos antibody (catalog #226–003, Synaptic Systems) at 1:1000 dilution with a 1:5000 dilution of mouse anti-mCherry antibody (catalog #632453, Clontech) overnight at 4°C. Alexa Fluor-conjugated secondary antibodies were used to detect primary antibody binding as described above. c-Fos as a marker for neural activity was quantified using a custom macro in FIJI (Schindelin et al., 2012).

### RNA extractions and quantitative PCR

Brains were extracted from male and female WT and heterozygous *Oprk1-Cre* mice (age range, ∼8–12 weeks) and flash frozen. Brains were homogenized using a Dounce homogenizer, and total RNA was isolated using the RNAEasy Midi Kit (Qiagen) following manufacturer instructions. RNA was treated with DNase (Ambion). Total RNA was quantified on a NanoDrop 1000 Spectrophotometer (Thermo Fisher Scientific). RNA (1 mg) was reverse transcribed using the High-Capacity Reverse Transcription Kit from Applied Biosystems. cDNA was diluted 1:5, and 2 µl of that solution were subjected to real-time PCR amplification to detect *Oprk1* (Mm01230885_m1, Thermo Fisher Scientific) with 5 µl of 1× SSoAdvanced Universal Probes Master Mix (BIO-RAD), 0.5 μl of 20× TaqMan Primer/Probe Mix (Applied Biosystems) in a total volume of 10 μl. Data were normalized to the endogenous control genes for transferrin (*Tfrc*; Mm00441941_m1, Thermo Fisher Scientific) or gusducin B (*GusB*; Mm019768_m1, Thermo Fisher Scientific).

### Western blotting

Heterozygous *Oprk1-Cre* and their WT littermates were killed by cervical decapitation, and their brains were rapidly removed and frozen in isopentane. Brains were sectioned at 300 µm thickness on the cryostat, and the amygdala (AMY, BLA+CeA) and NAc were dissected using a 2 mm biopsy punch. Tissue samples were homogenized in lysis buffer containing 320 mm sucrose, 5 mm HEPES, 1 mm EGTA, 1 mm EDTA, and 1% SDS, protease inhibitor cocktail (diluted 1:100), and phosphatase inhibitor cocktails II and III (diluted 1:100; Sigma-Aldrich) followed by brief sonication and then heating at 95°C for 5 min. Protein concentration was determined by a colorimetric Lowry assay (DC protein assay, BIO-RAD). Protein (20 µg) was resolved on a 10% SDS-polyacrylamide gel and electrophoretically transferred onto a polyvinylidene difluoride membrane. After the transfer, membranes were blocked with 5% nonfat dry milk (NFDM) in tris-buffered saline containing Tween-20 (TTBS) for 1 h at room temperature followed by overnight incubation at 4°C with primary antibody in 5% NFDM in TTBS, as shown in Extended Data Table 3-1. Membranes were washed in TTBS 3× for 10 min at 27°C, labeled with appropriate peroxidase-conjugated secondary antibodies (1:10,000 in TTBS; BIO-RAD), washed again 3× in TTBS, incubated in chemiluminescent reagent (Immobilon Crescendo Western HRP substrate, Millipore Sigma), and exposed to film. Membranes were stripped and reprobed using a primary antibody in 2.5% milk in TTBS for total protein levels for each protein target in Extended Data Table 3-1. Densiometric analyses was performed using FIJI. Phosphoprotein levels were normalized to total protein levels, and results were expressed as a percentage of the mean of the WT values for each gel to normalize data across blots.

### Striatal membrane preparation and [^35^S]GTPγS binding

Mouse striatum, including both ventral and dorsal striatum, were dissected from adult mice and frozen at −80°C. To prepare membranes, striatal tissue was homogenized using a Dounce homogenizer in cold homogenization buffer containing 10 mm Tris-HCl, pH 7.4, 100 mm NaCl, and 1 mm EDTA, as described previously (Bohn et al., 2015; Zhou et al., 2015). Homogenates were centrifuged at 20,000 × *g* for 30 min at 4°C. The resulting pellet was resuspended in homogenization buffer and incubated at 37°C for 30 min to remove endogenous opioids (McDonald and Lambert, 2010). The homogenate was centrifuged again at 20,000 × *g* for 30 min at 4°C, followed by resuspension in homogenization buffer and centrifugation. The resulting pellet was then resuspended in the assay buffer (10 mm Tris-HCl, pH 7.4, 100 mm NaCl, 1 mm EDTA, 5 mm MgCl_2_). Membrane protein concentration was determined using the BCA Protein Assay (catalog #23225, Thermo Fisher Scientific), followed by addition of 1 mm DTT (final concentration) to the membranes. Membranes were stored at −80°C for future analysis.

For [^35^S] GTPγS binding, striatal membrane proteins (2.5 µg) were incubated in the assay buffer containing 1 nm [^35^S] GTPγS, 10 μm GDP, and increasing concentrations of Dyn A (10 nm to 10 μm) for 1 h at 30°C in a total volume of 100 µl. The reactions were terminated by transferring reaction mixture onto GF/B filters using a 96-well plate harvester followed by three washes with ice-cold assay buffer. The membrane-bound [^35^S]GTPγS was retained on the filters, whereas free [^35^S]GTPγS was washed off. Filters were dried for a couple hours, and radioactivity was determined with a TopCount Microplate Scintillation Counter (PerkinElmer). Nonspecific binding was determined in the presence of 10 μm GTPγS, and the basal binding was determined in the absence of Dyn. Specific GTPγS binding was calculated by subtracting nonspecific activity from the total binding. Data were presented as a fold change over the basal activity. The sigmoidal Dyn dose–response curves were generated using the three-parameter nonlinear regression analysis. The values for EC_50_ and maximal stimulation (Emax) were extrapolated from the curves.

### Elevated plus maze

The elevated plus maze (EPM) consisted of two open and two closed arms perpendicular to each other. The maze was elevated ∼40 cm above the ground. The open arms measured 40 cm long × 5 cm wide. The closed arms were identical to the open arms but were enclosed by a 19-cm-high wall. The session was performed under dim white light. Mice were placed in the center of the maze facing the open arms and allowed to explore the maze for 5 min. The entire session was recorded using a video camera, and the results were scored manually by an observer blind to the genotype and experimental conditions. Parameters measured included the percentage of open arm entries as well as the percentage of time spent in open arms. For experiments examining the consequences of chemogenetic activation of BLA^KOR^ cells, all mice (mCherry-injected and hM3DQ-injected mice) were injected with the designer receptors exclusively activated by designer drug (DREADD) agonist CNO (3 mg/kg) 30 min before being placed on the elevated plus maze. Mice were tested using the same parameters used for baseline behavioral testing.

### Open field test

Mice were placed in a 43 × 43 cm open field chamber under dim white light for 10 min. The entire session was recorded using a video camera, and the results were analyzed using Biobserve Viewer III to determine the amount of time spent in the center and the periphery of the open field. *Oprk1-Cre* mice injected in the BLA with AAVs encoding either hM3DQ or mCherry were administered CNO (3 mg/kg, i.p.) 30 min before being placed in the open field chamber.

### Electronic von Frey

An electronic von Frey (eVF) apparatus (TopCat Metrology) was used to measure mechanical nociception. Mice were acclimated in elevated acrylic compartments (11 × 14 × 21.5 cm) on a mesh stand for at least 5 min before testing. Following this acclimation period, an eVF filament was applied to the mid-plantar region of the hindpaw, and withdrawal thresholds were recorded. The filament was applied to alternating left and right hindpaws at 3 min intervals for a total of two measurements per paw. The average score of these four tests served as the dependent measure.

### Thermal nociception tests

Thermal nociception was evaluated with a hot plate or cold plate apparatus, with a 20 × 20 cm metal surface maintained at either 54°C or 3°C and surrounded by a 26-cm-high Plexiglas wall. The nociceptive response was defined as the paw withdrawal latency in seconds. A sharp withdrawal, licking, or shaking of any hindpaw was considered a positive response.

### U-50488-induced conditioned place aversion and CNO conditioning

Mice were trained in a 27.3 × 27.3 cm^2^ open-field apparatus (Med Associates) equipped with two chambers that had different floor textures (rods and holes) and wall patterns (vertical and horizontal stripes). The two chambers were separated by a Plexiglas door that was open during the habituation and test sessions and closed during training sessions. Before training, drug-naive hM3DQ and control mCherry-injected mice were habituated to the apparatus and allowed access to both chambers for 15 min in a pretest session. Mice were administered 5 mg/kg (i.p.) CNO 30 min before the conditioning session followed by 2.5 mg/kg (i.p.) U-50488 15 min before the session. The study design was unbiased in that half of the mice received the drug in their preferred compartment, whereas half of them received the drug in their nonpreferred compartment. For CPA experiments, mice received two conditioning sessions per day. The first conditioning session was in the morning when mice received vehicle injections and were confined to one side of the chamber for 30 min. In the afternoon session, mice were injected with U-50488 and CNO and were confined to the other side of the chamber for 30 min. They received three vehicle and three CNO/U-50488 pairings. Twenty-four hours after the last conditioning session, mice were allowed access to both chambers for 15 min. A difference score was defined as the difference in the percentage of time spent in the drug (U-50488/CNO)-paired compartment postconditioning minus the time spent in the drug-paired compartment preconditioning. For experiments in WT mice, there were two groups. One group received saline on both sides of the compartment and another group received saline on one side and U-50488 (2.5 mg/kg) on the other side. Pretest, conditioning, and post-test sessions were conducted as described above.

We also examined whether activation of BLA^KOR^ cells was inherently rewarding or aversive using a place-conditioning test. CNO conditioning was done exactly as outlined above using an unbiased design. Mice received CNO (5 mg/kg, i.p.) and were confined to one side of the chamber for 30 min. The next day they received an equal volume of vehicle (10 ml/kg) and were confined to the opposite side. Mice were subjected to one conditioning session per day. They received four vehicle and four CNO pairings. Twenty-four hours after the last conditioning session, all mice were allowed access to both chambers for 15 min in a post-test session. A difference score was calculated as above.

### Three-chambered social interaction test

The three-chambered social interaction was performed in a Plexiglas box [60 (length) × 40 (width) × 22 (height) cm] composed of three chambers. Two empty inverted wire cups were placed in the outer chambers of the apparatus, and mice were acclimated to the apparatus for 5 min, 24 h before the start of the test. On the day of the test, mice were injected with CNO (5 mg/kg, i.p.), 30 min before the start of the test. An age-matched and sex-matched unfamiliar conspecific (target mouse) was placed under one of the wire cups. The wire cup in the other outer chamber was left empty. The side of the chamber that had the target mouse was alternated between the two outer chambers. A mouse was placed in the box for 10 min, and the amount of time that the mouse spent in each of the chambers was recorded. The entire session was recorded using a video camera, and time spent in each chamber was scored using Biobserve Viewer III. A sociability index was calculated for each mouse as (time spent in chamber with target mouse – time spent in chamber with empty cup)/(time spent in chamber with target mouse + time spent in chamber with empty cup).

### Electrophysiology

Mice were briefly (∼15–20 s) anesthetized with isoflurane, brains were rapidly removed, and placed in ice-cold choline buffer [(in mm) 25 NaHCO_3_, 1.25 NaH_2_PO_4_, 2.5 KCl, 0.5 CaCl_2_, 7 MgCl_2_, 25 d-glucose, 110 C_5_H_14_ClNO (choline chloride), 11.60 C_6_H_7_NaO_6_ (sodium ascorbate), and 3.09 C_3_H_3_NaO_3_ (sodium pyruvate)] for sectioning. Coronal slices, 300 μm thick, containing BLA were prepared using a VT1200s Leica Vibratome and transferred to artificial CSF (ACSF; 127 mm NaCl, 25 mm NaHCO_3_, 1.25 mm NaH_2_PO_4_, 2.5 mm KCl, 25 mm d-glucose, 2 mm CaCl_2_, 1 mm MgCl_2_) in a water bath for 30 min at 37°C. Slices then remained at room temperature until they were used for recordings. ACSF was continually perfused over the slice during recordings at a rate of ∼2 ml/min and maintained at ∼32–34°C, and slices were continuously aerated with a 5% carbon/95% oxygen mixture throughout preparation and recording. Slices and ACSF were replaced either after each recording or every hour between recordings to ensure optimal recording conditions.

Borosilicate glass pipettes (World Precision Instruments) with series resistances between 3 and 6 MΩ were fabricated using a horizontal puller (model P-97, Sutter Instruments). Pipette capacitance was compensated but series resistance was not. A potassium-based internal solution was used for all recordings (128 mm K-gluconate, 10 mm HEPES, 10 mm sodium phosphocreatine, 4 mm magnesium chloride, 4 mm sodium ATP, 0.4 mm sodium GTP, 3 mm ascorbic acid, 1 mm EGTA, and in some cases ∼3.5 mg/ml biocytin). Blockers were not used for any of the recordings. hM3DQ-labeled cells were visualized using an inverted microscope with 10× and 60× objectives (Olympus) coupled with a Prizmatix LED system (Prizmatix LTD). Series resistance for each neuron was recorded in voltage-clamp mode and was required to be <30 MΩ for inclusion in the analysis. Neuronal properties were recorded in current-clamp mode at resting membrane potential and at holding potential (by injecting additional current to maintain the cell at −70 mV). Frequency/current responses (duration, 1 s; amplitude, −200 to 200 pA; 20 pA steps) and spontaneous AP firing (current injection, 0 pA; for 3 min, sampled at 20 kHz) were also collected in current-clamp mode. All recordings were filtered and digitized at 10 kHz. Cells were allowed to equilibrate for 3–5 min before the onset of recordings and each cell was recorded from twice, once at baseline, and 10 min after the addition of 1 μm CNO (Hello Bio). All protocols were made and run using Clampex (version 11.2) and analyzed using Clampfit (version 10.7.0.3) software (Molecular Devices).

### Statistical analyses

All data are presented as the mean ± SEM. Statistical analysis was assessed using unpaired or paired *t* tests and two-way ANOVA. *Post hoc* Sidak tests were performed when a significant interaction was detected. Curve fit analyses were performed to analyze [^35^S]GTPγS binding data. Detailed statistical methods for each figure are provided in an attached table of statistics (Table 1).

**Table 1:** Table of summary statistics

Figure	Parameter	Type of test	Sample size	Statistical data
3*A*	[^35^S]GTPγS binding	Nonlinear curve fit	WT male = 7 *Cre/+* male = 7	EC_50_ WT = 1.33 × 10^–7^ M for WT and 1.268 × 10^−7^ for *Cre/+* *R*_2_ = 0.7756 for WT and 0.7597 *for Cre/+*
3*B*	P-ERK levels normalized to T-ERK	Unpaired *t* test Two tailed	WT female = 4, *Cre/+* female = 3, *Cre/+* male = 2	*t* = 0.68, df = 10, *p* = 0.51
3*C*	P-JNK levels to T-JNK	Unpaired *t* test, two-tailed	WT female = 4, *Cre/+* female = 3, Cre/+ male = 2	*t* = 0.498. df =7, *p* = 0.6335
3*D*	P-p38 levels normalized to total p38	Unpaired *t* test, two-tailed	WT female = 4, *Cre/+* female = 3, *Cre/+* male = 2	*t* = 0.043, df =10. *p* =0. 97
4*A*	Elevated plus maze, percentage open arm entries	Two-way ANOVA	WT male = 7, WT female = 12; *Cre/+* male = 8, *Cre/+* female = 7	*F*_genotype (1,30)_ = 0.21, *p* = 0.66 *F*_sex (1,30)_ = 4.2, *p* = 0.049 *F*_genotype × sex (1,30)_ = 0.32, *p* = 0.57
4*B*	Elevated plus maze, percentage open arm time	Two-way ANOVA	WT male = 7, WT female = 12; *Cre/+* male = 8, *Cre/+* female = 7	*F*_genotype (1,30)_ = 0.74, *p* = 0.4 *F*_sex (1,30)_ = 0.99, *p* = 0.33 *F*_genotype × sex (1,30)_ = 0.46, *p* = 0.50
4*C*	Percentage time in center, open field	Two-way ANOVA	WT male = 8, WT female = 11; *Cre/+* male = 9, *Cre/+* female = 7	*F*_genotype (1,31)_ = 1.97, *p* = 0.17 *F*_sex (1,31)_ = 4.4, *p* = 0.04 *F*_genotype × sex (1,31)_ = 0.00003, *p* = 0.99
4*D*	Mean withdrawal threshold, electronic von Frey	Two-way ANOVA	WT male = 8, WT female = 10; *Cre/+* male = 8, *Cre/+* female = 7	*F*_genotype (1,29)_ = 0.18, *p* = 0.68 *F*_sex (1,29)_ = 9.82, *p* = 0.0039 *F*_genotype × sex (1,29)_ = 3.78, *p* = 0.06
4*E*	Response latency, hot plate	Two-way ANOVA	WT male = 8, WT female = 10; *Cre/+* male = 8, *Cre/+* female = 7	*F*_genotype (1,29)_ = 1.28, *p* = 0.27 *F*_sex (1,29)_ = 3.76, *p* = 0.06 *F*_genotype × sex (1,29)_ = 2.58, *p* = 0.12
4*F*	Response latency, cold plate	Two-way ANOVA	WT male = 8, WT female = 9; *Cre/+* male = 9, *Cre/+* female = 7	*F*_genotype (1,29)_ = 0.09, *p* = 0.77 *F*_sex (1,29)_ = 1.7, *p* = 0.2 *F*_genotype × sex (1,29)_ = 1.94, *p* = 0.17
5*B*	Number of action potentials	Paired *t* test, two-tailed	Baseline = 7, CNO = 7	*t* = 3.08, df = 9, *p* = 0.022
5*C*	Resting membrane potential	Paired *t* test, two-tailed	Baseline = 7, CNO = 7	*t* = 3.757, df = 9, *p* = 0.0094
5*E*	c-Fos counts	Unpaired *t* test, two-tailed	Veh = 3, CNO = 4	*t* = 4.995, df = 5, *p* = 0.0041
7*A*	Elevated plus maze, percentage open arm entries	Two-way ANOVA	mCherry male = 8, mCherry female = 10; hM3DQ male = 8, hM3DQ female = 9	*F*_virus (1,31)_ = 6.64, *p* = 0.015 *F*_sex (1,31)_ = 2.51, *p* = 0.12 *F*_virus × sex (1,31)_ = 7.49, *p* = 0.01
7*B*	Elevated plus maze, percentage open arm time	Two-way ANOVA	mCherry male = 8, mCherry female = 10; hM3DQ male = 8, hM3DQ female = 9	*F*_virus (1,31)_ = 9.4, *p* = 0.005 *F*_sex (1,31)_ = 5.07, *p* = 0.032 *F*_virus × sex (1,31)_ = 12.22, *p* = 0.001
7*C*	Percentage time in center, open field	Two-way ANOVA	mCherry male = 9, mCherry female = 11; hM3DQ male = 9, hM3DQ female = 9	*F*_virus (1,34)_ = 1.12, *p* = 0.29 *F*_sex (1,34)_ = 0.03, *p* = 0.87 *F*_virus × sex (1,34)_ = 0.48, *p* = 0.49
7*D*	Sociability index	Two-way ANOVA	mCherry male = 9, mCherry female = 11; hM3DQ male = 9, hM3DQ female = 10	*F*_virus (1,35)_ = 0.48, *p* = 0.83 *F*_sex (1,35)_ = 0.01, *p* = 0.92 *F*_virus × sex (1,35)_ = 6.76, *p* = 0.01
8*A*	U-50488 CPA, difference score	Two-way ANOVA	mCherry male = 9, mCherry female = 10; hM3DQ male = 8, hM3DQ female = 11	*F*_virus (1,34)_ = 1.656, *p* = 0.21 *F*_sex (1,34)_ = 0.016, *p* = 0.89 *F*_virus × sex (1,34)_ = 4.67, *p* = 0.037
8*B*	CNO CPP, difference score	Two-way ANOVA	mCherry male = 5, mCherry female = 7; hM3DQ male = 6, hM3DQ female = 7	*F*_virus (1,21)_ = 0.0004, *p* = 0.98 *F*_sex (1,21)_ = 0.85 *p* = 0.37 *F*_virus × sex (1,21)_ = 1.005, *p* = 0.3276
Extended Data 3-1*A*	Relative *Oprk1*expression, whole brain	Unpaired *t* test, two-tailed	WT female = 5 *Cre/+* female = 5	*t* = 7.43, df = 8, *p* < 0.0001
Extended Data 3-1*B*	Relative *Oprk1*expression, NAc	Unpaired *t* test, two-tailed	WT male = 4 WT female = 2 *Cre/+* male 3 = *Cre/+* female = 5	*t* = 2.934, df = 12, *p* = 0.013
Extended Data 3-2*A*	Maximal G-protein stimulation	Unpaired *t* test, two-tailed	WT male = 7 *Cre/+* male = 7	*t* = 0.12, df = 12, *p* = 0.9
Extended Data 3-2*B*	EC_50_	Unpaired *t* test, two-tailed	WT male = 7 *Cre/+* male = 7	*t* = 0.07, df = 12, *p* = 0.95
Extended Data 3-2*C*	Basal [^35^S]GTPγS binding	Unpaired *t* test, two-tailed	WT male = 7 *Cre/+* male = 7	*t* = 0.22, df = 12, *p* = 0.83
Extended Data 3-3*A*	Amygdala p-ERK/T-ERK	Unpaired *t* test, two-tailed	WT female = 5, *Cre/+* male = 2 male, *Cre/+* female = 2	*t* = 0.712, df = 7, *p* = 0.499
Extended Data 3-3*B*	Amygdala p-JNK/T-JNK	Unpaired *t* test, two-tailed	WT female = 5, *Cre/+* male = 2 male, *Cre/+* female = 2	*t* = 0.715, df = 7, *p* = 0.498
Extended Data 3-3*C*	Amygdala P-p38/T-p38	Unpaired *t* test, two-tailed	WT female = 5, *Cre/+* male = 2 male, *Cre/+* female = 2	*t* = 1.922, df = 6, *p* = 0.1030
Extended Data 4-1	Number of closed arm entries, WT vs *Oprk1-Cre*	Two-way ANOVA	mCherry male = 7, mCherry female = 12; hM3DQ male = 8, hM3DQ female = 7	*F*_genotype (1,30)_ = 1.335, *p* = 0.26 *F*_sex (1,30)_ = 0.76, *p* = 0.39 *F*_genotype × sex (1,30)_ = 0.66, *p* = 0.43
Extended Data 7-1	Number of closed arm entries, mCherry vs hM3DQ	Two-way ANOVA	mCherry male = 8, mCherry female = 10; hM3DQ male = 8, hM3DQ female = 9	*F*_virus (1,31)_ = 0.02, *p* = 0.88 *F*_sex (1,31)_ = 0.43, *p* = 0.52 *F*_virus × sex (1,31)_ = 0.62, *p* = 0.43
Extended Data 8-1	Difference score percentage (Post-Pre)	Unpaired *t* test	WT male = 8–10	*t* = 2.322, df =18, *p* = 0.032

P, Phosphorylated; T, total.

## Results

We used a variety of techniques to determine whether *Cre* expression was localized exclusively to *Oprk1* cells in the *Oprk1-Cre* mouse. We examined colocalization between *Cre* recombinase and *Oprk1* mRNA in the following three brain regions that express high amounts of *Oprk1*: BLA, claustrum/dorsal endopiriform nucleus, and the PVT. We found a high degree of colocalization between *Oprk1* and *Cre* transcripts in all of the brain regions examined in homozygous *Oprk1-Cre* mice. In the BLA (Fig. 1*A*), we found that 89% of *Oprk1* transcripts were colocalized with *Cre*. Similarly, we found 95% colocalization in the claustrum/dorsal endopiriform nucleus (Fig. 1*B*) and ∼91.1% colocalization in the PVT (Fig. 1*C*). These results indicate a high degree of fidelity of *Cre* expression to *Oprk1* cells in these brain regions.

**Figure 2.**
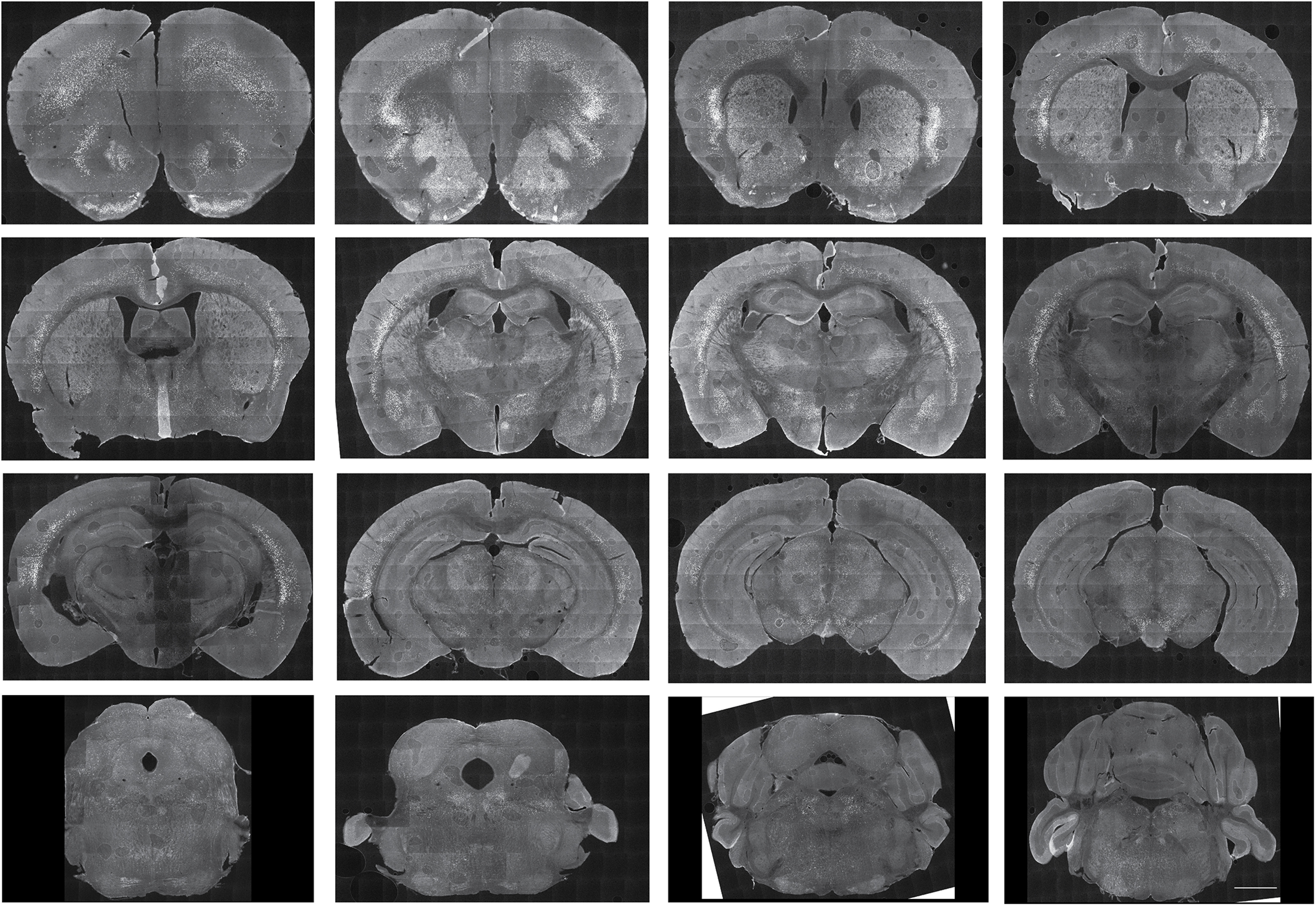
Brain-wide snapshots of Cre expression in the adult *Oprk1-Cre* mouse. Tiled images of Cre protein expression at 20× resolution across the brain of an adult *Oprk1-Cre* mouse are shown. Strongest Cre expression was observed in the claustrum and dorsal endopiriform nucleus. Cre expression was also observed in the PVT, CeA, BLA, PVT, DR, and locus coeruleus (LC). The pattern of Cre expression matched that of *Oprk1* expression in the Allen Brain Atlas. Scale bar, 1 mm. Please see Extended Data Figure 2-1 for distribution of BLA^KOR^ cells and Extended Data Figure 2-2 for colocalization between virally delivered Cre-dependent *EGFP* and *Oprk1* in the BLA.

To get a snapshot of Cre protein expression in the adult brain, we performed Cre IHC in adult homozygous *Oprk1-Cre* mice using a previously validated (Kellendonk et al., 1999) antibody against Cre recombinase. We found the highest Cre-like immunoreactivity in the claustrum and dorsal endopiriform cortex area. We also found Cre-immunoreactive cells in the dorsal and ventral striatum, the CeA, BLA, PVT, VTA, dorsal raphe (DR), and the locus coeruleus (Fig. 2). The pattern of Cre-like immunoreactivity closely mimicked the expression pattern of *Oprk1* mRNA in the Allen Brain Atlas.

10.1523/ENEURO.0043-23.2023.f2-1 Figure 2-1 Distribution BLA^KOR^ cells. GFP expression was examined in *Oprk1-Cre*:: L10A-EGFP mice. GFP expression was distributed throughout the BLA in anterior sections (left). This expression pattern shifted medially and ventrally in more posterior sections. This expression pattern matched the pattern found on the Allen Brain Atlas (right). Scale bar, 200 µm. The numbers shown in panels on the right correspond to image numbers in the Allen Brain Atlas for *Oprk1* mRNA expression (coronal sections). Download Figure 2-1, TIF file.

10.1523/ENEURO.0043-23.2023.f2-2 Figure 2-2 Colocalization between *EGFP*-expressing and *OPRK1*-expressing cells in the BLA. We examined colocalization between *Oprk1* and virally delivered Cre-dependent *EGFP* expression in the BLA of *Oprk1-Cre* mice. ***A***, Representative images showing *EGFP* and *Oprk1* expression in the BLA. ***B***, Quantification of the results are shown and revealed that 93% of *EGFP*-expressing cells also expressed *Oprk1*. *N* = 3 female mice. Scale bar, 100 µm. Download Figure 2-2, TIF file.

We also crossed *Oprk1-Cre* mice with the floxed L10A-EGFP reporter mouse (Krashes et al., 2014) to determine the distribution of KOR cells within the BLA. In this mouse, EGFP is expressed in cells that express Cre recombinase during development and in adulthood. We found an interesting pattern of Cre expression in the BLA of these mice. In the anterior subdivisions of the BLA, EGFP expression, which reports Cre expression, was distributed uniformly throughout the BLA. Expression shifted to medial and ventral aspects of the BLA in more posterior subdivisions. This pattern of Cre expression closely mirrored the *Oprk1* expression found in the Allen Brain Atlas (Extended Data Fig. 2-1).

We also determined colocalization between virally delivered Cre-dependent *EGFP* and *Oprk1* mRNA expression in the BLA using *in situ* hybridization. Our results revealed that 93% of EGFP-expressing cells also expressed *Oprk1* (Extended Data Fig. 2-2). These results indicate high specificity of viral-mediated labeling of *Oprk1* cells in the BLA.

To determine whether the insertion of Cre recombinase into the 3′UTR of the *Oprk1* locus altered *Oprk1* expression, we performed qPCR on whole brain and NAc mRNA from WT and heterozygous *Oprk1-Cre* mice (henceforth referred to as *Oprk1-Cre* mice). We found an ∼0.6-fold increase in *Oprk1* mRNA expression in *Oprk1-Cre* mice compared with WT mice (Extended Data Fig. 3-1*A*). We also found a similar increase in *Oprk1* mRNA expression in the NAc (Extended Data Fig. 3-1*B*).

**Figure 3.**
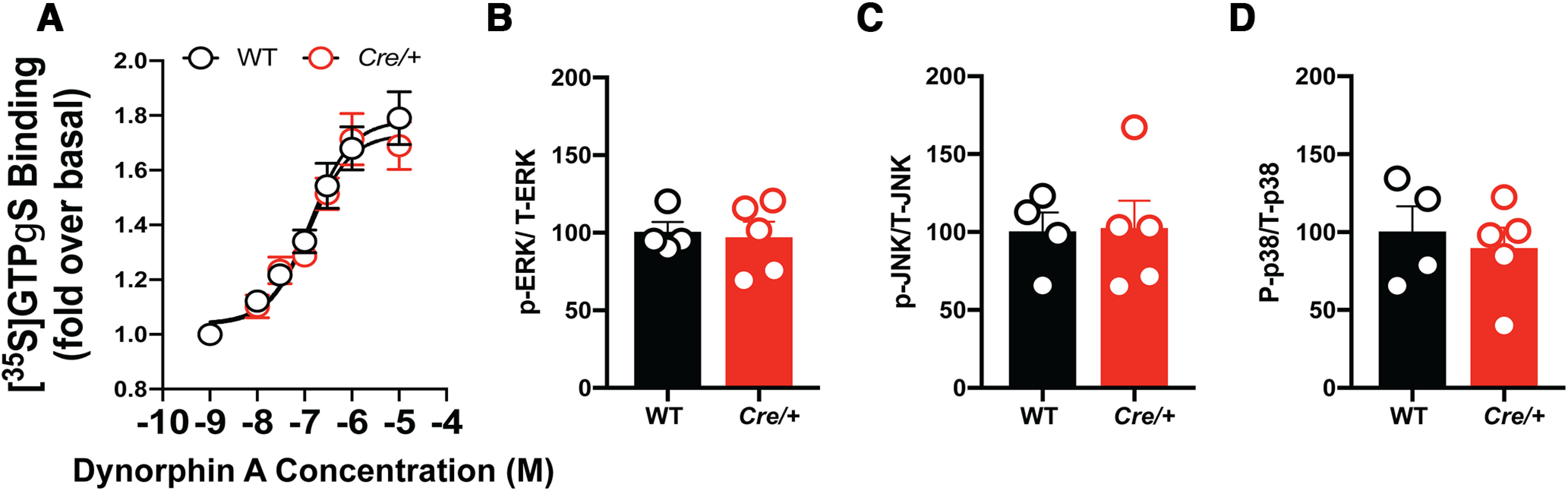
KOR function is intact in *Oprk1-Cre* mice. ***A***, Dyn-stimulated GTPγS binding did not differ significantly between WT and *Oprk1-Cre* mice. *n* = 7 males/group. ***B–D***, No differences were observed in phosphorylated (P)-ERK (***B***), P-JNK (***C***), and P-p38 (***D***) in the NAc under basal conditions. *N* = 4–5/group (WT = 4 females, Cre/+ = 3 females and 2 males). Please see Extended Data Figure 3-1 for *Oprk1* mRNA expression in WT and *Oprk1-Cre* mice; Extended Data Figure 3-2 for [^35^S]GTPγS binding in the striatum of WT and *Oprk1-Cre* mice; and Extended Data Figure 3-3 for basal KOR signaling is not altered in the amygdala of *Oprk1-Cre* mice. Extended Data Table 3-1 lists antibodies used for Western blotting.

To determine whether this apparent increase in *Oprk1* mRNA levels translates to changes in KOR function, we examined Dyn-induced [^35^S]GTPγS binding in striatal membranes prepared from WT and *Oprk1-Cre* mice (Bohn et al., 2015; Zhou et al., 2015; Massaly et al., 2019). Dyn-stimulated GTPγS binding was similar in both genotypes (Fig. 3*A*). Basal, Emax, and EC_50_ values for [^35^S] GTPγS binding (fmole/µg protein) also did not differ between genotypes (Extended Data Fig. 3-2*A–C*). Therefore, KOR–G-protein coupling was not altered in *Oprk1-Cre* mice.

10.1523/ENEURO.0043-23.2023.f3-1 Figure 3-1 ***Oprk1* mRNA expression in WT and *Oprk1-Cre* mice.** We measured *Oprk1* mRNA levels in WT and *Cre/+* mice by quantitative PCR. *Oprk1* mRNA levels (relative to *Gapdh* and *Tfrc*) were significantly increased in (***A***) whole brain mRNA from *Cre/+* mice (*, *p* < 0.001, *N* = 5 female mice/group). ***B***) *Oprk1* expression was also increased in the NAc of *Cre/+* mice (*, *p* = 0.0125, WT male = 4, WT female = 2, *Cre/+* male = 3, *Cre/+* female = 5). Download Figure 3-1, TIF file.

10.1523/ENEURO.0043-23.2023.f3-2 Figure 3-2 [^35^S]GTPγS binding in the striatum of WT and *Oprk1-Cre* mice. ***A–C***, WT and heterozygous *Oprk1-Cre* mice did not differ in maximal stimulation in response to agonist over basal activity (***A***), EC_50_ values of Dyn to activate G-protein (***B***), and basal GTPγS binding (***C***). *N* =7 males/group. Download Figure 3-2, TIF file.

10.1523/ENEURO.0043-23.2023.f3-3 Figure 3-3 Basal KOR signaling is not altered in the amygdala of *Oprk1-Cre* mice. ***A–C***, Basal phosphorylation levels of ERK (***A***), JNK (***B***), and p38 (***C***) were not different between WT and *Oprk1-Cre* mice in the amygdala (WT female = 5; *Cre/+* male = 2, *Cre/+* female = 2). Download Figure 3-3, TIF file.

10.1523/ENEURO.0043-23.2023.tab3-1 Table 3-1 List of antibodies used with catalog numbers and dilutions. Download Table 3-1, DOCX file.

We also examined downstream components of KOR signaling in the NAc (Fig. 3*B–D*) and amygdala (Extended Data Fig. 3-3*A–C*) of *Oprk1-Cre* mice. KOR activation increases phosphorylation of ERK, JNK, and P38 kinases (Bruchas et al., 2006; Al-Hasani and Bruchas, 2011). We reasoned that if the observed change in *Oprk1* mRNA expression led to a functional change in KOR signaling, we would see an increase in phosphorylation of these downstream effectors. We found no differences in basal phosphorylation levels of ERK, JNK, and P38 kinases in the NAc (Fig. 3*B–D*) or amygdala (Extended Data Fig. 3-3*A–C*) between *Oprk1-Cre* mice and their WT littermates, suggesting that the increase in *Oprk1* mRNA did not lead to a functional increase in KOR signaling at baseline.

**Figure 4.**
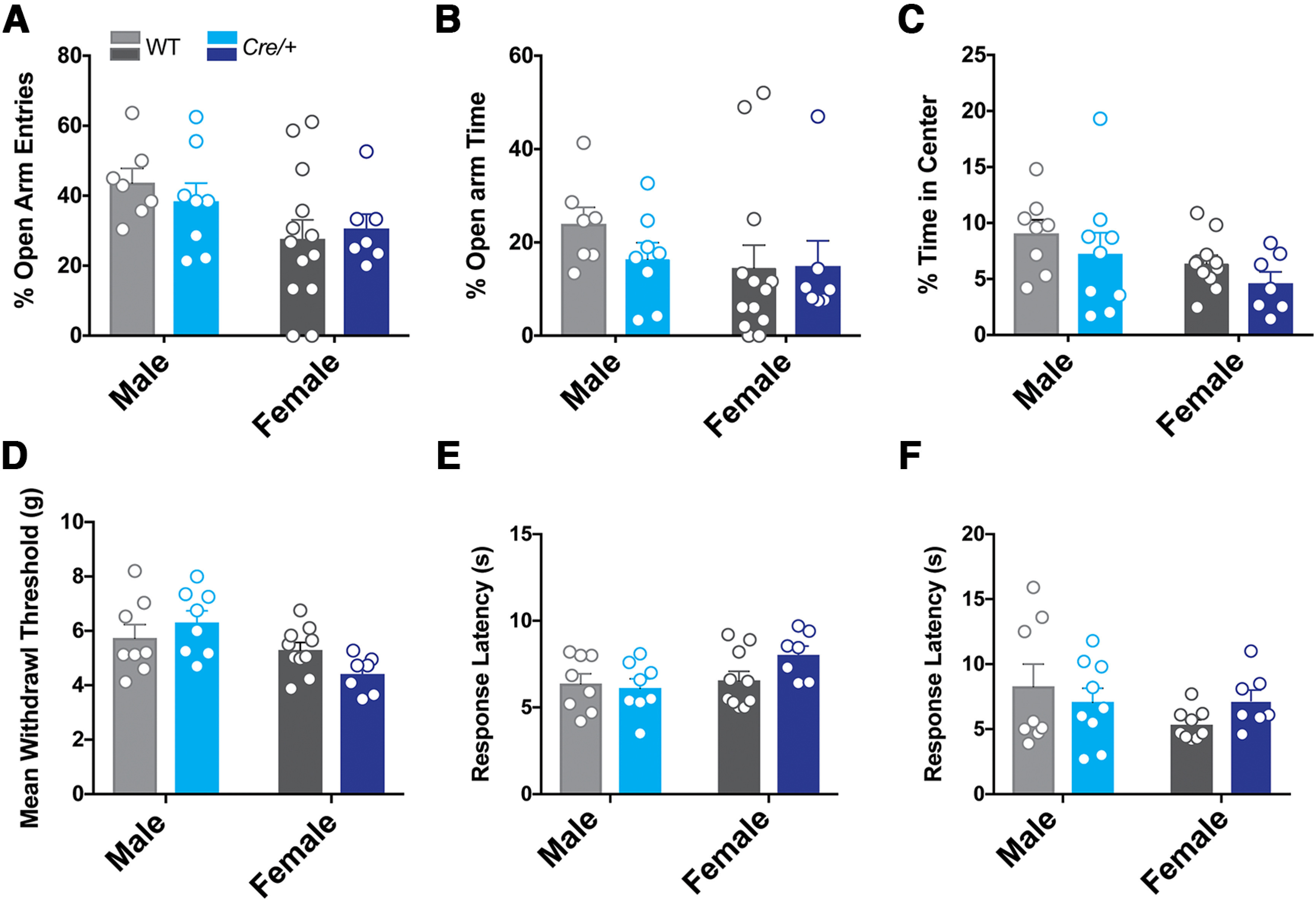
Baseline anxiety-like and pain behaviors in *Oprk1-Cre* mice. ***A*–*C***, Anxiety-like behaviors were tested using the EPM and open field anxiety tests in WT and *Oprk1-Cre* mice. There were no significant genotypic differences in open arm entries (***A***) or time (***B***) in the EPM (WT male = 7, WT female = 12; *Cre/+* male = 8, *Cre/+* female = 7). ***C***, There were also no genotypic differences in the percentage of time in the center of the open field (WT male = 8, WT female = 11; *Cre/+* male = 9, *Cre/+* female = 7). ***D***, There were no genotype differences seen in the mean withdrawal threshold in the eVF mechanical nociception test (WT male = 8, WT female = 10; *Cre/+* male = 8, *Cre/+* female = 7) and response latencies (***D***) on the hot plate tests (***E***; WT male = 8, WT female = 10; *Cre/+* male = 8, *Cre/+* female = 7) and cold plate tests (***F***) and (WT male = 8, WT female = 9; *Cre/+* male = 9, *Cre/+* female = 7). See Extended Data Figure 4-1 for closed arm entries were not altered on the EPM in *Oprk1-Cre* mice.

**Figure 5.**
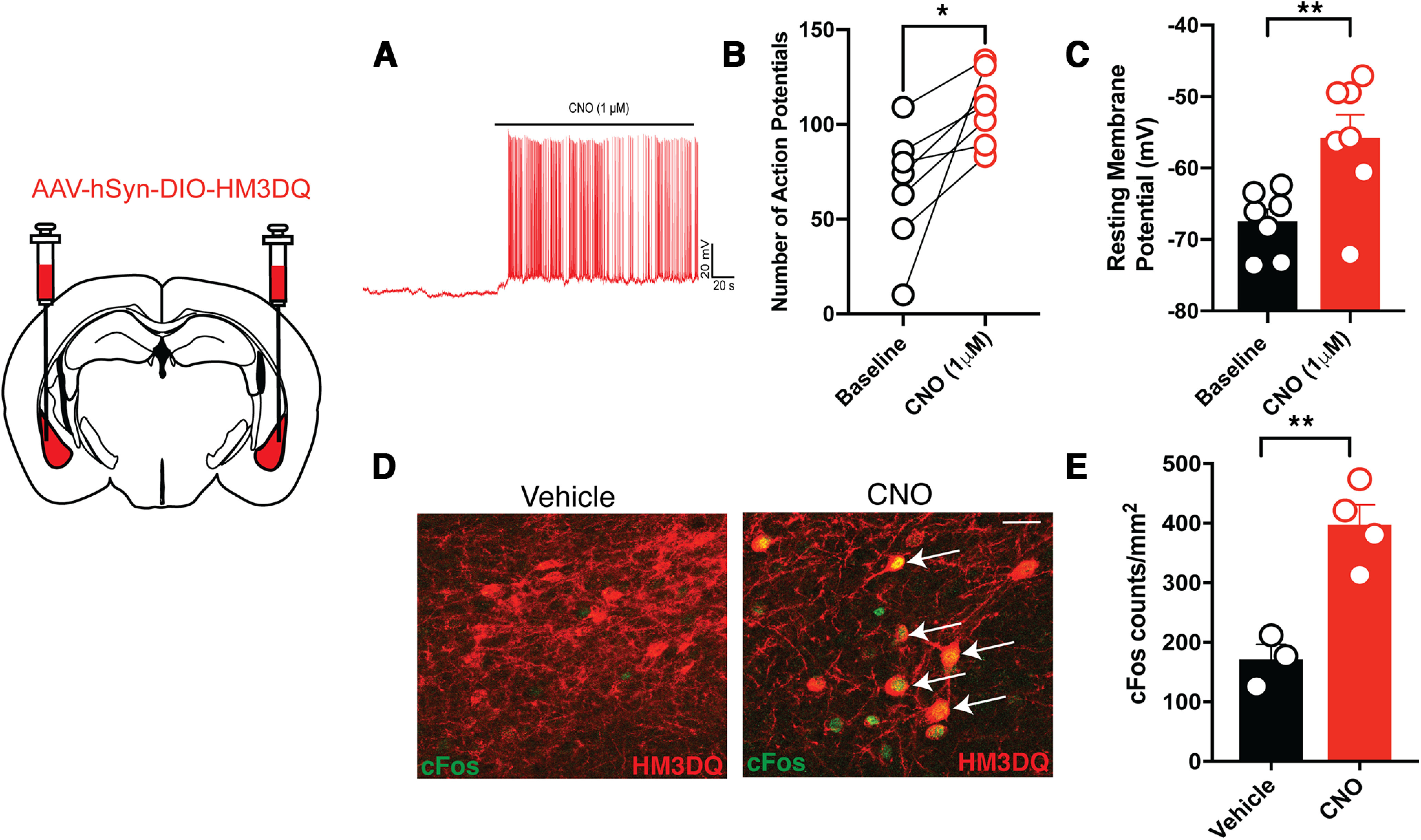
Validation of DREADD-induced activation of BLA^KOR^ neurons. ***A*–*C***, CNO application increased both spontaneous and electrically evoked excitability in BLA^KOR^ cells expressing hM3DQ. *p = 0.0217, **p = 0.0091, paired *t* test, *n* = 7 cells/group. ***D***, Visual representation of c-Fos activation in the BLA of hM3DQ-injected *Oprk1-Cre* mice treated with vehicle or CNO before killing. ***E***, Quantification of c-Fos shows a significant increase in the activation in CNO-injected mice compared with vehicle-injected mice. **p = 0.0041, unpaired *t* test, *N* =3–4 mice/group.

We next examined whether there were baseline differences in behaviors that are known to be modulated by KOR signaling. First, we examined anxiety-like behaviors using EPM and open field tests. For the EPM, we measured the percentage of open arm entries and the percentage of open arm time (Fig. 4*A*,*B*). Two-way ANOVA of the percentage of open arm entries showed a main effect of sex (*F*_sex (1,30)_ = 12.08, *p* = 0.0493) with females venturing less into the open arms than their male counterparts. However, there was no genotype or genotype × sex interaction (Fig. 4*A*). Analysis of the percentage of open arm time did not reveal a main effect of sex, genotype, or genotype × sex interaction (Fig. 4*B*). Analysis of the number of closed arm entries also did not reveal main effects of sex, genotype, or sex × genotype interaction (Extended Data Fig. 4-1). We next examined the percentage of time spent in the center of an open field (Fig. 4*C*). Two-way ANOVA revealed a main effect of sex (*F*_sex (1,31)_ = 4.4, *p* = 0.04) with females spending less time exploring the center of the open field. However, we did not detect the main effects of genotype or genotype × sex interaction. Next, we examined nociceptive sensitivity in these mice. Since the activation of KORs has analgesic effects (Snyder et al., 2018), we compared nociceptive thresholds between WT and *Oprk1-Cre* mice under basal conditions. We used the electronic von Frey test to measure mechanical nociception and hot and cold plate tests to measure thermal nociception. Two-way ANOVA of mean withdrawal pressure in the eVF test revealed a main effect of sex (*F*_sex (1,21)_ = 9.82, *p* = 0.004), with females displaying lower mean withdrawal thresholds than males (Fig. 5*D*). However, there were no main effects of genotype or genotype × sex interaction. Two-way ANOVA of mean withdrawal latencies on the hot plate and cold plate tests did not reveal significant main effects of sex, genotype, or genotype × sex interaction (Fig. 4*E*,*F*). In summary, we did not detect genotypic differences in any of the baseline behaviors tested.

10.1523/ENEURO.0043-23.2023.f4-1 Figure 4-1 Closed arm entries were not altered on the EPM in *Oprk1-Cre* mice. No genotype or sex differences were found in the number of closed arm entries between male and female WT and *Oprk1-Cre* mice. *N* = 7–10/group for males and *N* = 7–12/group for females. Download Figure 4-1, TIF file.

Since optogenetic stimulation of BLA inputs to the BNST is anxiolytic (Crowley et al., 2016), we predicted that chemogenetic activation of BLA^KOR^ cells would reduce anxiety-like behaviors. We validated DREADD-induced activation of BLA^KOR^ neurons by electrophysiology using amygdala brain slices from *Oprk1-Cre* mice expressing hM3DQ. We found that bath application of CNO (1 μm) significantly increased both spontaneous and evoked firing of BLA^KOR^ cells expressing hM3DQ (Fig. 5*B–D*). We also found that intraperitoneal administration of 3 mg/kg CNO significantly increased c-Fos expression in the BLA of hM3DQ-expressing mice compared with mice administered vehicle as a control (Fig. 5*E*).

**Figure 6.**
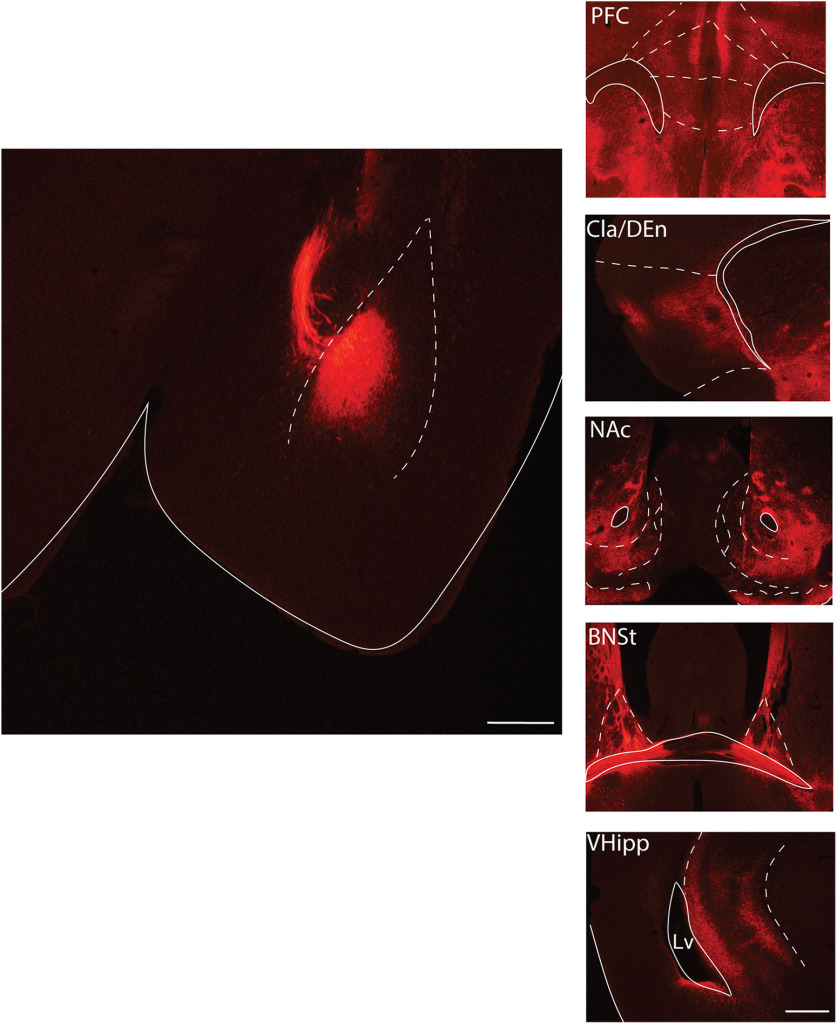
Projections of BLA^KOR^ neurons. Image of mCherry injection into the BLA (left). BLA^KOR^ neurons project to the prefrontal cortex (PFC), the claustrum/dorsal endopiriform nucleus, the NAc, the bed nucleus of the stria terminalis (BNST), and the ventral hippocampus (VHipp; right).

We also examined projection targets of BLA^KOR^ cells. We injected Cre-dependent mCherry into the BLA of *Oprk1-Cre* mice and examined mCherry fluorescence throughout the brain. We found strong projections from BLA^KOR^ cells to the mPFC, lateral subdivision of the NAc, claustrum/dorsal endopiriform cortex, and the ventral hippocampus (Fig. 6).

**Figure 7.**
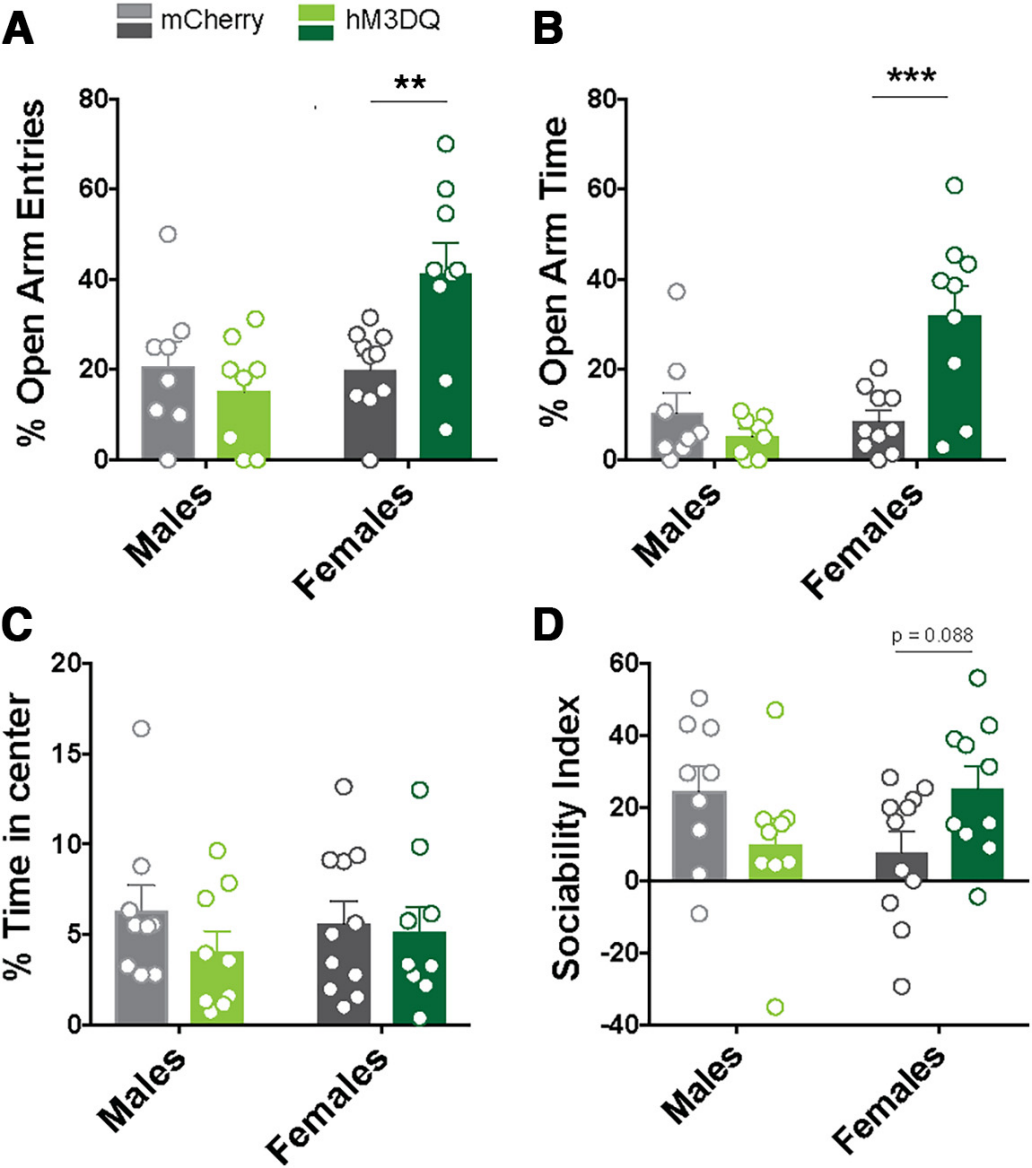
Chemogenetic activation of BLA^KOR^ cells and anxiety-like behavior. ***A***, There was no significant difference between mCherry and hM3DQ-injected male mice in open arm entries or open arm time in males (*N* = 8/group). ***B***, There was a significant increase in the percentage of open arm entries (**p = 0.0016) and open arm time (***p = 0.0010, Sidak post-test) in hM3DQ-injected female mice compared with mCherry controls (*N* =9–10 mice/group). Please see Extended Data Figure 7-1 for closed arm entries in mCherry-injected and hM3DQ-injected mice. ***C***, There were no differences in the time spent in the center of an open field in male or female hM3DQ-injected mice compared with mCherry controls (mCherry male = 9, mCherry female = 11; hM3DQ male = 9, hM3DQ female = 9). ***D***, There was no difference in sociability index in male mCherry-injected and hM3DQ-injected mice. Female hM3Dq mice showed increased sociability compared with mCherry controls. This result did not reach statistical significance (*p* = 0.088, Sidak post-test; mCherry male = 9, mCherry female = 11; hM3DQ male = 9, hM3DQ female = 10).

To determine whether the activation of BLA^KORs^ affects anxiety-like behaviors, mice were administered CNO (3 mg/kg) 30 min before the EPM and open field tests. Mice were first tested on the EPM. Two-way ANOVA of the percentage of open arm entries revealed a significant virus × sex interaction (*F*_virus × sex (1,31)_ = 7.491, *p* = 0.0102]. *Post hoc* Sidak test revealed that hM3DQ-injected females made significantly more open arm entries than mCherry controls (*p* = 0.0162; Fig. 7*A*). Two-way ANOVA of the percentage of time spent in open arms also revealed a significant virus × sex interaction *F*_virus × sex (1,31)_ = 12.22, *p* = 0.0014). The hM3DQ-injected females spent significantly more time in the open arms than mCherry controls (*p* = 0.0010; Fig. 6*B*). There were no significant differences in the percentage of open arm entries or the percentage of open arm time between mCherry and hM3DQ-injected males (Fig. 7*A*,*B*). These results indicate an anxiolytic phenotype in hM3DQ-expressing female mice. There were no significant differences in the number of closed arm entries among any of the groups tested (Extended Data Fig. 7-1). Mice were also tested for anxiety-like behaviors on the open field 24 h after the EPM test. Two-way ANOVA of the percentage of time spent in the center of the open field did not reveal any significant main effects of virus, sex, or an interaction between the two (Fig. 7*C*). No differences were observed between the groups in total distance traveled in the open field (data not shown), suggesting that locomotor activity was not impaired by CNO injections. We next tested the effects of BLA^KOR^ activation on sociability using the three-chamber social interaction test. Two-way ANOVA of the sociability index revealed a significant virus × sex interaction (*F*_genotype × sex(1,35)_ = 6.764, *p* = 0.0135; Fig. 7*D*). *Post hoc* analyses did not reveal any significant differences between mCherry-injected and hM3DQ-injected male mice. Female hM3DQ-injected mice displayed increased sociability compared with mCherry controls; however, this result did not reach statistical significance (*p* = 0.088).

10.1523/ENEURO.0043-23.2023.f7-1 Figure 7-1 Closed arm entries were not altered on the EPM in mCherry-injected and hM3DQ-injected mice. The number of closed arm entries was not significantly different between mCherry-injected and hM3DQ-injected male and female mice. *N* = 8/group for males and *N* = 9–12/group for females. Download Figure 7-1, TIF file.

**Figure 8.**
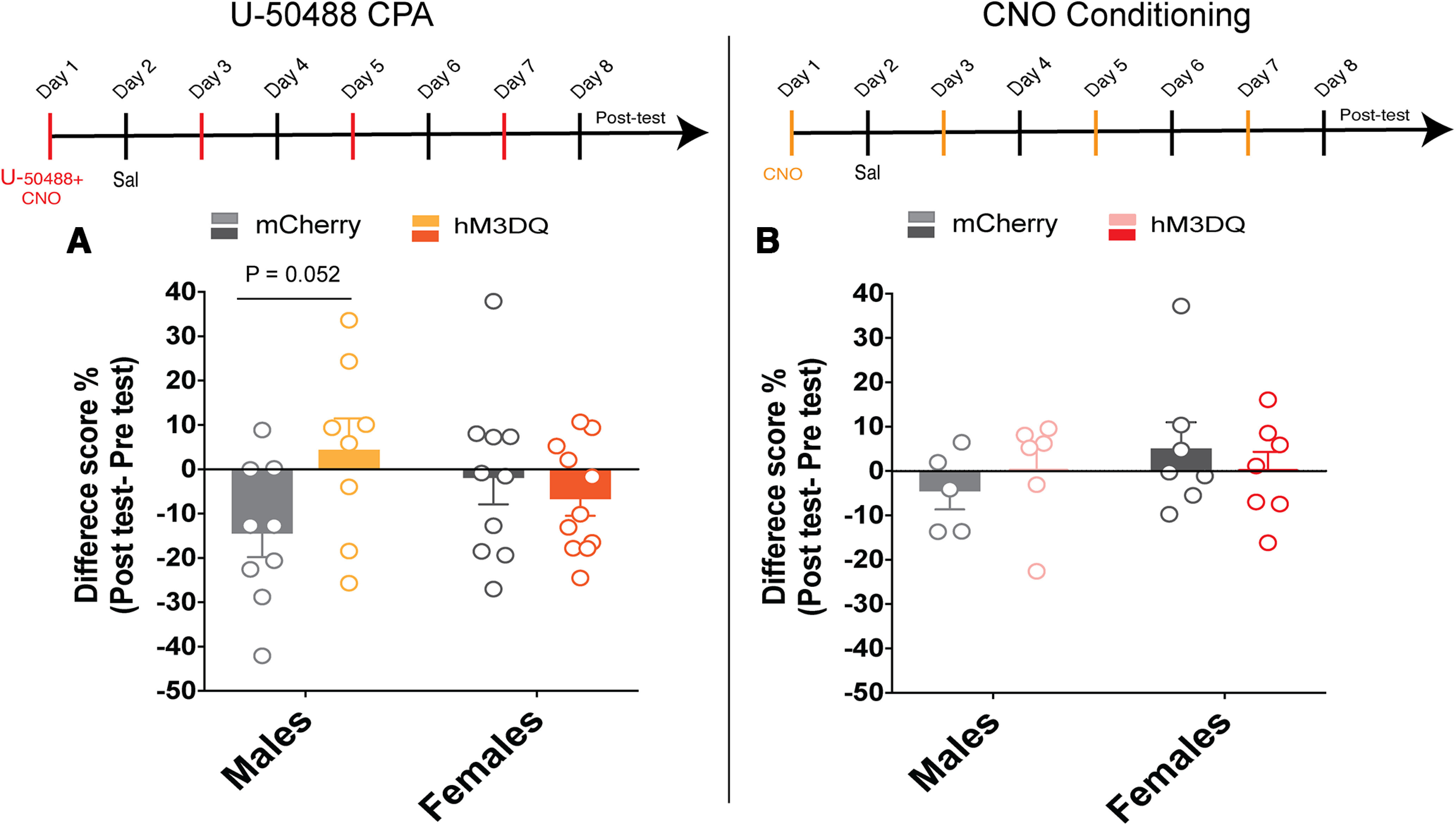
Chemogenetic activation of BLA^KOR^ cells attenuates U-50488 induced CPA. ***A***, CNO administration attenuated U-50488-induced CPA in hM3DQ-injected males compared with mCherry controls. However, this effect did not reach statistical significance (*p* = 0.0524, Sidak post-test; *N* = 8–9/group) in males and in females (*N* = 9–11/group). ***B***, Neither male nor female hM3DQ-injected *Oprk1-Cre* mice form a preference for or avoid the CNO-paired chamber. *N* = 5–6/group for males and *N* = 5–7/group for females. See Extended Data Figure 8-1 for U-50488-induced CPA in C57BL/6J mice.

KOR agonists are aversive presumably because of KOR-mediated inhibition of target cells (Tejeda and Bonci, 2019). To determine whether BLA^KORs^ contribute to this effect, we sought to counteract KOR inhibition by chemogenetically activating BLA^KOR^ cells. Mice expressing hM3DQ or mCherry were administered vehicle injections and were then confined to one side of the conditioned place preference (CPP) apparatus. Later the same day they received the injections of KOR agonist U-50488 (2.5 mg/kg) and CNO (5 mg/kg) and were confined to the other side. Preference for the drug-paired side before and after conditioning was determined and a difference score was calculated. Two-way ANOVA analysis of the difference scores revealed a significant virus × sex interaction (*F*_virus × sex (1,34)_ = 4.671, *p* = 0.0378]. Injections of U-50488 plus vehicle produced CPA in mCherry-injected male, but not female, *Oprk1-Cre* mice. CPA was attenuated in U-50488 plus CNO-injected males that expressed hM3DQ; however, this result did not reach statistical significance (*p* = 0.0524; Fig. 8*A*). In contrast to males, female mice expressing mCherry or hM3DQ failed to develop CPA (Fig. 8*B*). Since the magnitude of CPA observed in mCherry-injected *Oprk1-Cre* males is lower (∼15%) than what has been reported in the literature (Land et al., 2009), we decided to examine U-50488-induced CPA in C57BL/6J (WT) mice (Extended Data Fig. 8-1). We compared WT mice that received saline injections on both sides of the apparatus to those that that received saline on one side and U-50488 (2.5 mg/kg) on the other side. We found that mice that received U-50488 injections showed significant CPA to the drug-paired side compared with mice that received saline on both sides of the chamber (**p < 0.05, unpaired *t* test). Importantly, the magnitude of this CPA (∼12%) was similar to that observed in mCherry-injected *Oprk1-Cre* mice.

10.1523/ENEURO.0043-23.2023.f8-1 Figure 8-1 U-50488-induced CPA in C57BL/6J mice. Systemic injection of 2.5 mg/kg U-50488 produced CPA in WT mice. Difference score obtained by subtracting the percentage of time spent in drug-paired compartment before and after conditioning is shown for saline-injected and U-50488-injected mice. U-50488-injected mice showed CPA compared with mice that received saline injections on both sides of the chamber. *p = 0.032, *t* test, *N* =8–10 male mice/group. Download Figure 8-1, TIF file.

We next determined whether the activation of BLA^KOR^ cells was inherently reinforcing by measuring the development of CPP after the administration of CNO (5 mg/kg). We observed no significant differences in the percentage of time spent in the CNO-paired compartment before and after conditioning (Fig. 8*C*,*D*). Our results indicate that chemogenetic activation of BLA^KOR^ cells by itself is neither aversive nor rewarding. Together, these results suggest that KOR-mediated inhibition of BLA^KOR^ cells contributes to the aversive effects of KOR agonists in males, but not in females.

## Discussion

Here we describe the generation and characterization of a transgenic mouse line that expresses Cre recombinase under the control of the *Oprk1* promoter. This mouse was generated to provide a tool for the visualization and manipulation of KOR-expressing neurons throughout the brain and periphery. Using a variety of methods including IHC, *in situ* hybridization, and virally delivered reporter gene expression, we have shown that Cre recombinase expression in this mouse line is restricted to KOR-expressing cells throughout the brain and that KOR function is preserved in *Oprk1-Cre* mice. We also show functional validation of this mouse line by demonstrating that chemogenetic activation of BLA^KOR^ neurons can modulate anxiety-like behaviors, KOR agonist-induced conditioned aversion, and sociability.

This mouse line differs from the previously published *Oprk1-Cre* line in that the Cre recombinase is inserted into 3′UTR of the KOR locus. In the *Oprk1-Cre* line generated by the Ross laboratory (Cai et al., 2016), the Cre transgene is inserted into the second exon. Therefore, heterozygous Cre mice might be expected to express 50% less KOR than WT littermates. *Oprk1-Cre* mice described in this study show a 0.6-fold increase in *Oprk1* mRNA in several brain regions. We do not know the mechanism for this increase in *Oprk1* mRNA, although one possibility is that insertion of the Cre transgene in the 3′UTR of the *Oprk1* locus may have disrupted an unknown endogenous regulatory element. We investigated whether this change in *Oprk1* mRNA was associated with a functional change in KOR signaling and baseline behaviors known to be sensitive to modulation by KORs. Despite higher levels of mRNA expression, we found no differences in Dyn-stimulated GTPγS binding in striatal membranes prepared from control and *Oprk1-Cre* mice. We also examined signaling events occurring downstream of KORs including the activation of ERK, JNK, and p38 (Bruchas et al., 2006; Al-Hasani and Bruchas, 2011). We found no differences in the phosphorylation status of key downstream effectors of KOR signaling in the amygdala (CeA plus BLA) and NAc of *Oprk1-Cre* mice compared with WT mice under basal conditions. One caveat with the interpretation of these results is that changes in KOR signaling may be masked under basal conditions and should be examined after KOR agonist treatment to confirm that downstream signaling pathways remain intact in these mice. We also found no differences in baseline nociceptive thresholds and anxiety-like behaviors. Together, these results suggest that the increase in *Oprk1* mRNA did not lead to increased KOR function. We observed this change in *Oprk1* expression despite using a smaller P2A self-cleaving sequence instead of IRES between the KOR coding region and Cre. Changes in expression of an endogenous gene have been reported for other Cre knock-in lines where the Cre transgene has been inserted into the 3′UTR. For example, the DAT-Flpo (Kramer et al., 2021) and the DAT-IRES-Cre lines (Bäckman et al., 2006), in which Cre and Flpo transgenes are inserted int the 3′UTR of the DAT locus, both show decreases in DAT expression. Because there are no good commercially available antibodies specific for KOR, the expression of KOR proteins was not directly determined in our mouse line. Regardless, our data reveal that KOR function is unaltered, suggesting that this *Oprk1-Cre* line could be used for a variety of experiments designed to probe the function and anatomy of KOR cells in the brain.

Using RNA *in situ* hybridization, we have shown that *Cre* expression is localized to *Oprk1*-expressing cells in the following four brain regions: the BLA, claustrum and dorsal endopiriform cortex, and the PVT. We also found that the pattern of Cre protein expression in *Oprk1-Cre* mice closely resembled endogenous *Oprk1* mRNA expression in the brain. Further, we found a high degree of colocalization between virally delivered Cre-dependent *EGFP* and endogenous *Oprk1* expression in the BLA of *Oprk1-Cre* mice. Based on these results, we conclude that Cre is expressed with high fidelity in KOR-expressing cells. However, researchers interested in using this mouse line will need to confirm this with their chosen Cre-dependent virus or viruses in their brain region of interest.

To demonstrate the utility of these mice in exploring causal relationships between specific KOR cell populations in the brain and behavior, we decided to target KOR-expressing cells in the BLA. We first functionally validated DREADD expression in the BLA by examining neuronal firing of hM3DQ-expressing cells. We found that CNO application significantly enhanced the firing rate of BLA^KOR^ cells expressing hM3DQ. CNO application also increased c-Fos expression in hM3DQ-expressing BLA^KOR^ cells. CNO injection also resulted in sex-specific effects on the EPM with females displaying reduced anxiety-like behavior. This result is consistent with previous work showing that optogenetic activation of BLA neurons that project to BNST is anxiolytic (Crowley et al., 2016). By contrast, we did not observe any change in anxiety-like behavior in males. Since BLA^KOR^ cells project to many different brain regions, it is possible that selective activation of BNST-projecting BLA^KOR^ cells would have resulted in an anxiolytic phenotype in males. We also observed a trend toward increased sociability after chemogenetic activation of BLA^KOR^ cells in female, but not in male, *Oprk1-Cre* mice. Sex differences have been reported in the role of the Dyn/KOR system in stress reactivity and KOR agonist-induced aversion (Russell et al., 2014; Becker and Chartoff, 2019). Future studies will determine whether these sex differences emerge from changes in receptor density or localization in BLA terminal regions.

We also measured anxiety-like behavior in the open field and failed to observe any significant effects of BLA^KOR^ neuron activation on time spent in the center. One possible reason for this discrepancy with our EPM results could be that the open field is a more anxiogenic environment than the EPM, and indeed control mCherry-injected mice only spent ∼5% of their time in the center of the open field. Perhaps, higher doses of CNO are required to induce anxiolysis in such highly anxiogenic environments.

Dyn-mediated activation of KORs has been implicated in the aversive effects of stress. Similarly, pharmacological activation of KORs also results in dysphoria and aversion in rodents as well as in humans (Bruchas et al., 2010; Al-Hasani and Bruchas, 2011; Darcq and Kieffer, 2018). KOR-mediated inhibition of serotonergic neurons in the DRN and dopaminergic neurons in the VTA are implicated in mediating the aversive effects of KOR agonists and stress (Land et al., 2009; Bruchas et al., 2011). However, the contribution of BLA^KORs^ in the aversive effects of KOR activation remain unknown. Our results revealed that U-50488 treatment induced CPA in males but not females and that hM3DQ-mediated activation of BLA^KOR^ cells attenuated U-50488-induced CPA in males. These results implicate a role for KORs expressed in the BLA in regulating KOR agonist-mediated aversion, although we did not include a control group injected with vehicle+U-504888. Further, the magnitude of U-50488-induced CPA was similar in both male *Oprk1-Cre* and WT C57BL/6J mice suggesting intact in vivo KOR agonist efficacy in *Oprk1-Cre* mice. BLA^KOR^ cells project to the mPFC, NAc, ventral hippocampus, and insular cortex. Future studies will determine which of these downstream projection targets of BLA^KOR^ encode this aversion. Surprisingly, we failed to observe U-50488-induced CPA in females. One possible reason for this could be that higher doses of U-50488 are required to produce CPA in females.

We next investigated whether the activation of BLA^KOR^ cells themselves was rewarding. Our CNO place conditioning results reveal that repeated activation of BLA^KOR^ cells did not produce conditioned place preference or aversion. Optogenetic stimulation of BLA inputs to the NAc is highly rewarding and promotes self-stimulation (Stuber et al., 2011). By contrast, optical activation of BLA cell bodies was highly variable in supporting self-stimulation, suggesting that BLA regulates reward and reinforcement in a projection-specific manner. In summary, these results suggest that the inhibition of BLA^KOR^ cells by KOR agonists may in part underlie the aversive effects of KOR agonists.

In summary, we have generated a transgenic Cre-driver mouse line that expresses Cre recombinase under the control of the KOR promoter. In this mouse, Cre is expressed with very high fidelity in KOR cells. This mouse could be an important tool that allows for mapping the projections of KOR cells and dissecting the contribution of KOR circuits throughout the brain in addiction, pain, and stress-related behaviors.

## Synthesis

Reviewing Editor: Christie Fowler, University of California, Irvine Decisions are customarily a result of the Reviewing Editor and the peer reviewers coming together and discussing their recommendations until a consensus is reached. When revisions are invited, a fact-based synthesis statement explaining their decision and outlining what is needed to prepare a revision will be listed below. The following reviewer(s) agreed to reveal their identity: Hugo Tejeda, Sarah Ross.

This manuscript describes the development and characterization of a KOR-cre allele. Moreover, through manipulation of KOR-expressing neurons in the BLA, the authors reveal important sex-dependent differences. Specifically, activation of KOR neurons in the BLA reduces anxiety, as indicated by the elevated plus maze, in females but not in males. This sex-dependent effect is robust and unexpected. In contrast, only males show conditioned place aversion to a kappa agonists, and this effect is reversed upon activation of KOR neurons in the VTA. Overall, this is an outstanding study that describes extensive validation of a new genetic tool, high quality data, and several important new observations.

Upon our review consultation, it was concluded that additional experiments are not necessary, and the reviewer comments can be addressed within the manuscript text for a revision. Each reviewer’s concerns are as follows:

Reviewer 1:

The present study focuses on the development and basic characterization and utilization of KOR Cre mice. The authors demonstrate that Cre is expressed in KOR mRNA expressing cells. The authors also demonstrate use Cre-dependent vectors to map putative amygdala KOR-expressing neurons throughout the brain. The authors show that KOR expression is elevated but also demonstrate that Dyn-mediated G protein signaling was unaffected and basal levels of pERK, pJNK, and p-p38. Subsequently the authors employ behaviroral studies to demonstrate the utility of this line in in-vivo studies focused on KOR neurons of the amygdala. Overall this is a valuable resource for the field. Unfortunately, the characterization of the mouse line does not thoroughly test the specificity of the line in various domains, including ensuring that Cre-dependent viral-mediated labeling is specific to KOR-positive neurons, showing that agonists effects on signaling and/or function in-vivo or with other ex-vivo approaches are unaffected or not, and resolving whether some of the non-significant trends is true evidence that this mouse provides functional access to these cells based on KOR expression. Discussion of the limitations and providing readers some guidelines what they would need to do to characterize specificity when using this KOR-Cre line is warranted if not addressed experimentally. Below are additional comments aimed at being useful suggestions to increase the strengths of the present work.

1. For in-vivo experiments have the authors considered an inhibitory DREADD experiment which may mimic the effect of KOR signaling in KOR-positive neurons?

2. The authors should check the accuracy of the line depicting CNO application on their electrophysiology results. The cell starts firing even before the line.

3. The non-significant trends in sociability and CPA with the DREADD experiment should be noted as NS in the graphs to avoid misleading readers into thinking that it is significant. The authors also talk extensively about the non-significant data in the discussion which should be avoided. Increasing the sample size is necessary to truly see if this is a real effect.

4. The failure for a U50 mediated-CPA to develop in females, and the weak CPA seen in males (relative to previous reports with KOR agonists from many labs), leaves open the possibility of whether KOR agonist in vivo effects are modified in this KOR Cre line. Measures comparing these effects against WT controls would be useful for determining that. In the discussion (page 22 paragraph 3) the authors note a caveat by not including a control group but it should be specified that the genotype is what needs to be controlled for.

5. The biochemical analysis of downstream signaling though useful does not directly tie to KOR function under basal conditions. Changes in KOR signaling may be masked under basal conditions. The authors should temper their interpretation and discuss this caveat or conduct studies with drug challenge to truly show that KOR agonist induced signaling is not perturbed. This also holds true for the behavior as stated above.

6. The title should be modified to faithfully state what the mouse line provides. This does not include the localization and function of the KORs, but rather the cells that putatively express this molecule.

7. On page 5, paragraph 2, sentence 2 the authors should cite the paper they are referring to.

Reviewer 2:

This is an outstanding study that describes extensive validation of a new genetic tool, high quality data, and several important new observations.

Comments:

1. In the abstract, the authors write that activation of KOR neurons in the BLA led to increased anxiety-like behavior. I believe they mean decreased.

2. It is not clear from the methods whether experiments performed on male and female mice were performed in parallel, or whether experiments using cohorts of males and cohorts of females were performed on separate days. If the latter, it is more difficult to attribute differences in outcomes from cohort 1 and cohort 2 to sex with certainty, because there could be a large number of unknown variables that might change between the two sets of experiments. Please clarify this detail in the methods and/or results section. If experiments with males and females were performed on different dates, please note this limitation in the discussion.

3. In the results and texts, the authors say that activation of BLA-KOR cells attenuated KOR-agonist induced conditioned place aversion (CPA) in male but not in female Oprk1-Cre mice. I think this statement is somewhat misleading. I think a clearer description would be that kappa agonist induced conditioned place aversion was observed in male, but not female mice. The CPA observed in males was prevented by activation of KOR BLA neurons. In females, which did not show CPA to a kappa agonist, activation of KOR BLA neurons in the BLA during conditioning had no significant effect.

4. When describing a previously generated KOR-cre allele, the authors say “Therefore, heterozygous Cre mice express 50% less KOR than WT littermates.”Given that the message level in these mice has not, to my knowledge, been measured, I think it would be more appropriate to say “Therefore, heterozygous Cre mice might be expected to express 50% less KOR than WT littermates.” (As revealed in this very study, genetic manipulations can have unexpected effects on gene expression!)
