# Supplementary figures and images for: A Cre Driver Line for Genetic Targeting of Kappa Opioid Receptor Expressing Cells
Source: eNeuro. 2023 Jul 11;10(7):ENEURO.0043-23.2023. doi: 10.1523/ENEURO.0043-23.2023 (PMC10348446; doi:10.1523/ENEURO.0043-23.2023)

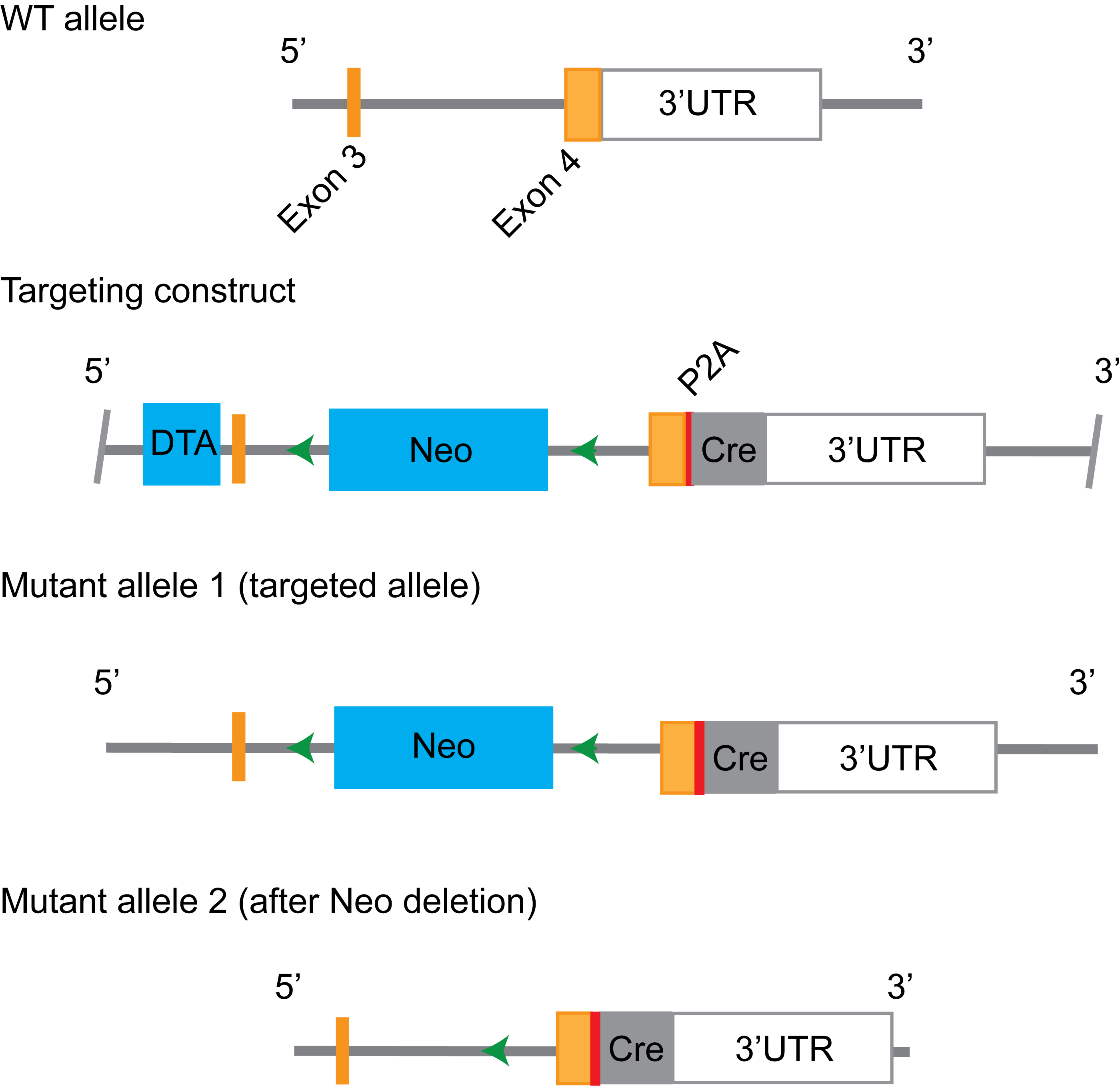

Supplement: Figure 1-1 — Schematic illustrating insertion of Cre recombinase into the 3′UTR of the KOR locus. Download Figure 1-1, TIF file. [file enu-eN-MNT-0043-23-s03.tif]

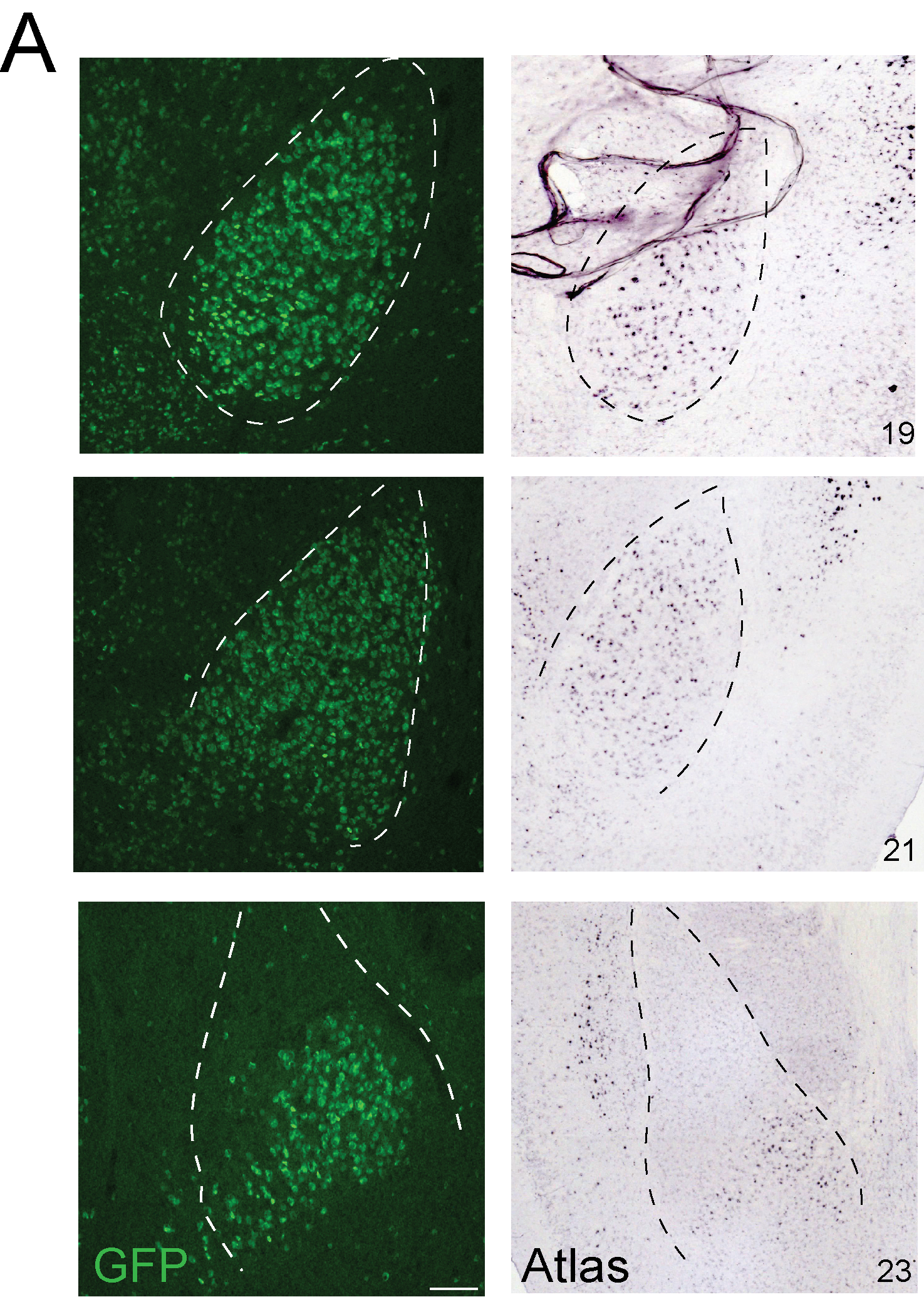

Supplement: Figure 2-1 — Distribution BLAKOR cells. GFP expression was examined in Oprk1-Cre:: L10A-EGFP mice. GFP expression was distributed throughout the BLA in anterior sections (left). This expression pattern shifted medially and ventrally in more posterior sections. This expression pattern matched the pattern found on the Allen Brain Atlas (right). Scale bar, 200 µm. The numbers shown in panels on the right correspond to image numbers in the Allen Brain Atlas for Oprk1 mRNA expression (coronal sections). Download Figure 2-1, TIF file. [file enu-eN-MNT-0043-23-s04.tif]

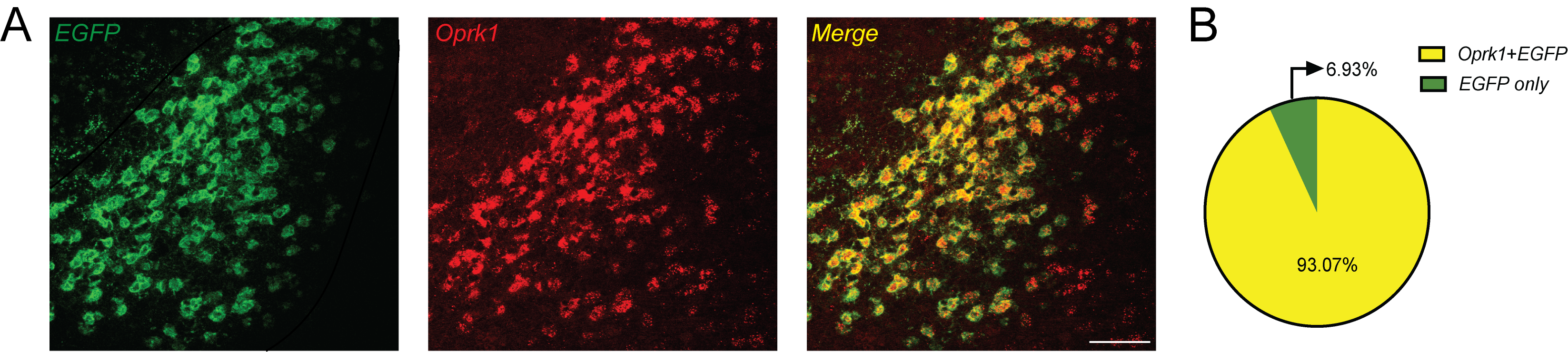

Supplement: Figure 2-2 — Colocalization between EGFP-expressing and OPRK1-expressing cells in the BLA. We examined colocalization between Oprk1 and virally delivered Cre-dependent EGFP expression in the BLA of Oprk1-Cre mice. A, Representative images showing EGFP and Oprk1 expression in the BLA. B, Quantification of the results are shown and revealed that 93% of EGFP-expressing cells also expressed Oprk1. N = 3 female mice. Scale bar, 100 µm. Download Figure 2-2, TIF file. [file enu-eN-MNT-0043-23-s05.tif]

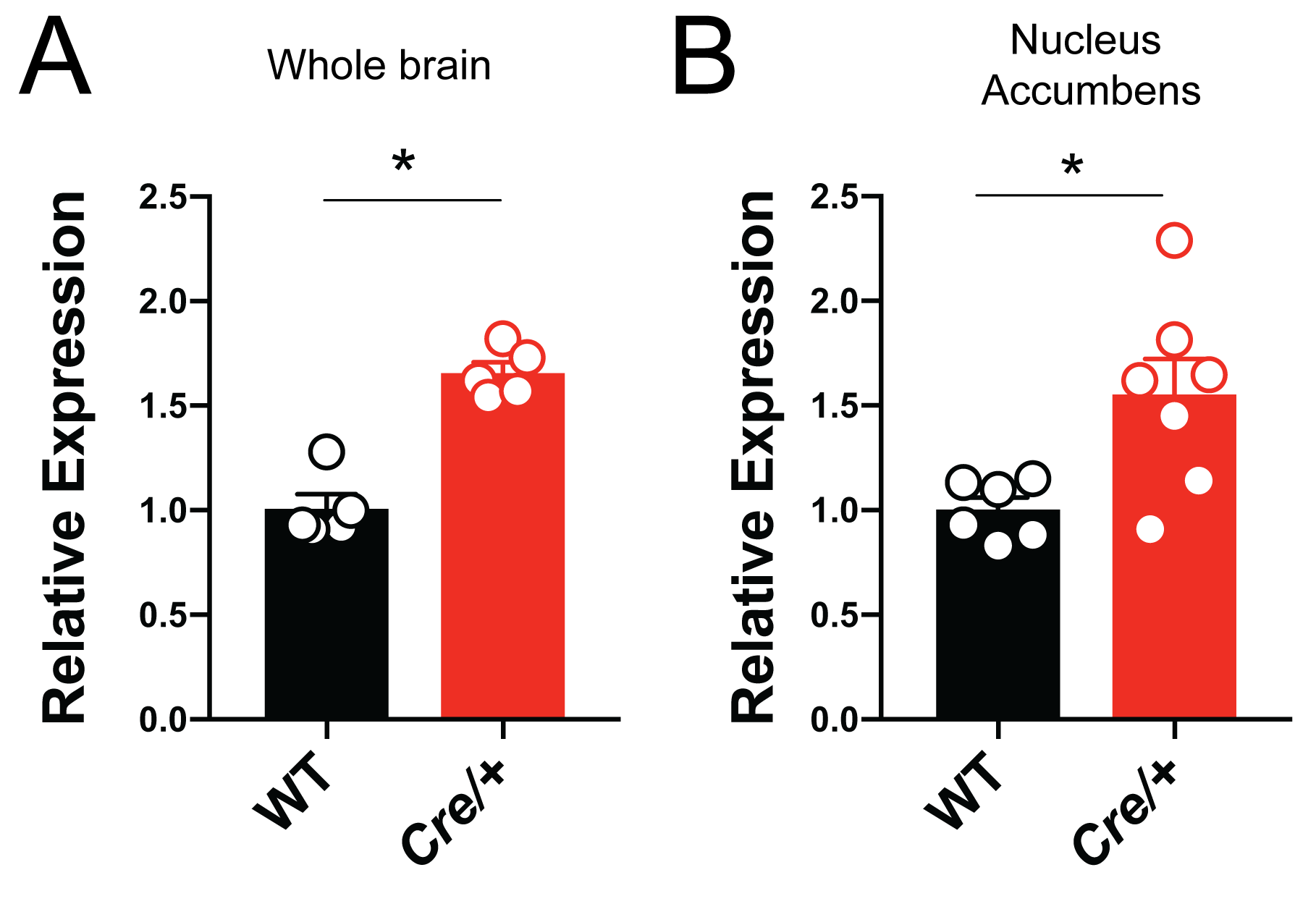

Supplement: Figure 3-1 — Oprk1 mRNA expression in WT and Oprk1-Cre mice. We measured Oprk1 mRNA levels in WT and Cre/+ mice by quantitative PCR. Oprk1 mRNA levels (relative to Gapdh and Tfrc) were significantly increased in (A) whole brain mRNA from Cre/+ mice (*, p < 0.001, N = 5 female mice/group). B) Oprk1 expression was also increased in the NAc of Cre/+ mice (*, p = 0.0125, WT male = 4, WT female = 2, Cre/+ male = 3, Cre/+ female = 5). Download Figure 3-1, TIF file. [file enu-eN-MNT-0043-23-s06.tif]

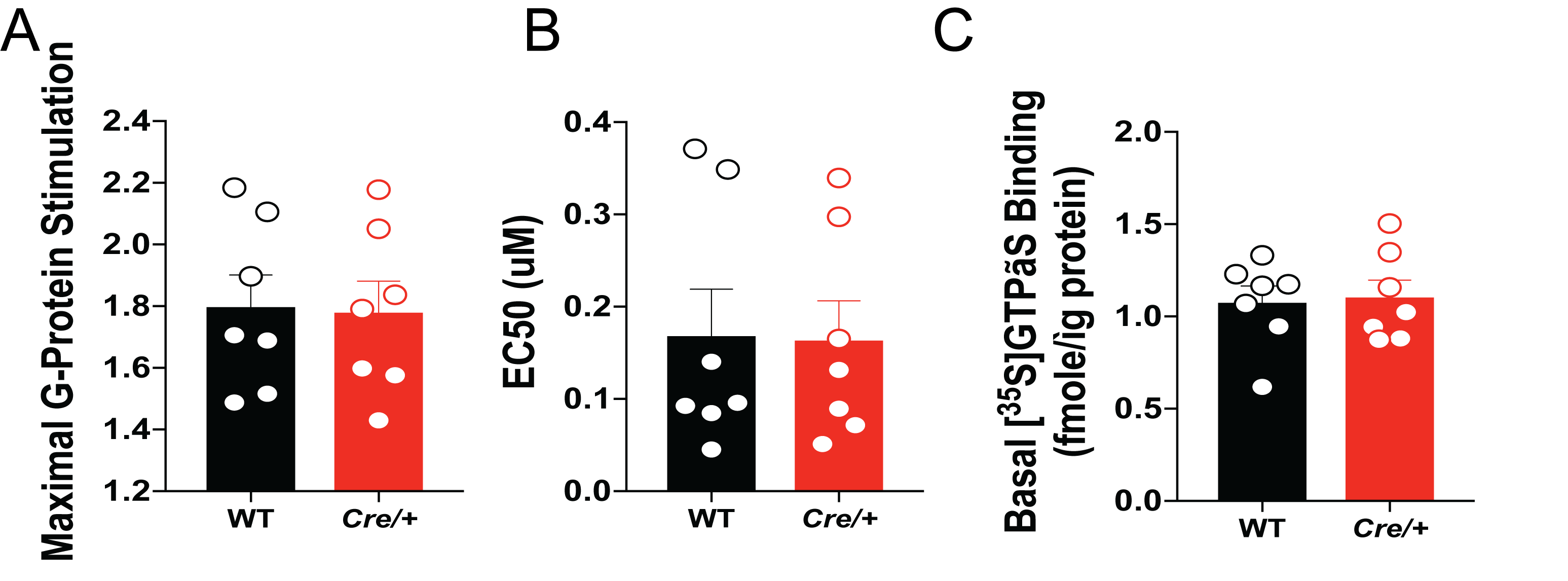

Supplement: Figure 3-2 — [35S]GTPγS binding in the striatum of WT and Oprk1-Cre mice. A–C, WT and heterozygous Oprk1-Cre mice did not differ in maximal stimulation in response to agonist over basal activity (A), EC50 values of Dyn to activate G-protein (B), and basal GTPγS binding (C). N =7 males/group. Download Figure 3-2, TIF file. [file enu-eN-MNT-0043-23-s07.tif]

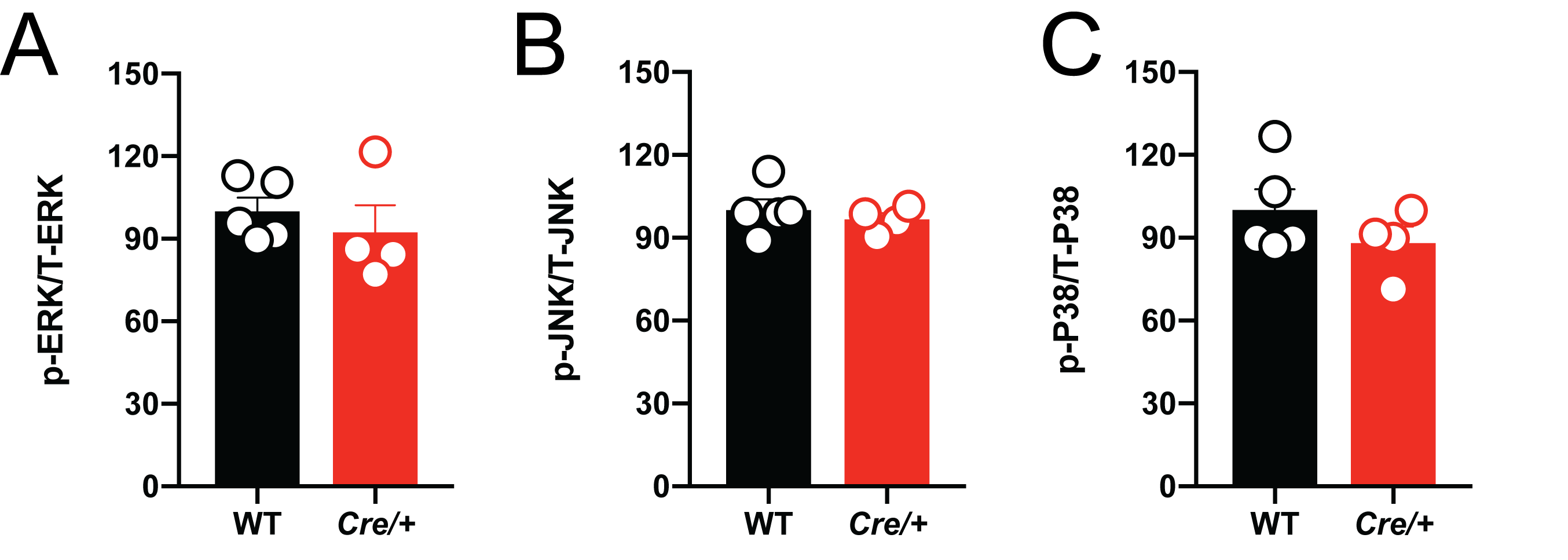

Supplement: Figure 3-3 — Basal KOR signaling is not altered in the amygdala of Oprk1-Cre mice. A–C, Basal phosphorylation levels of ERK (A), JNK (B), and p38 (C) were not different between WT and Oprk1-Cre mice in the amygdala (WT female = 5; Cre/+ male = 2, Cre/+ female = 2). Download Figure 3-3, TIF file. [file enu-eN-MNT-0043-23-s08.tif]

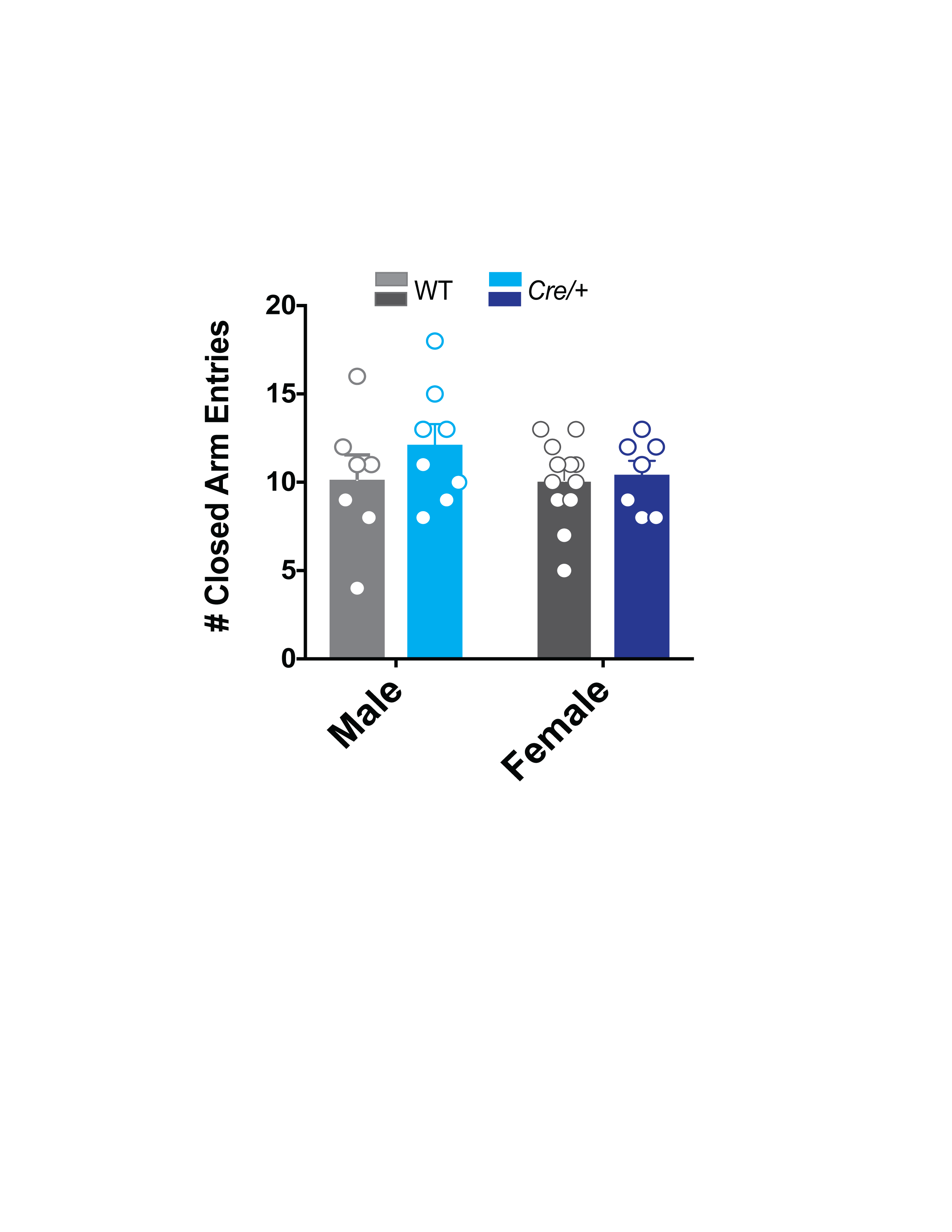

Supplement: Figure 4-1 — Closed arm entries were not altered on the EPM in Oprk1-Cre mice. No genotype or sex differences were found in the number of closed arm entries between male and female WT and Oprk1-Cre mice. N = 7–10/group for males and N = 7–12/group for females. Download Figure 4-1, TIF file. [file enu-eN-MNT-0043-23-s09.tif]

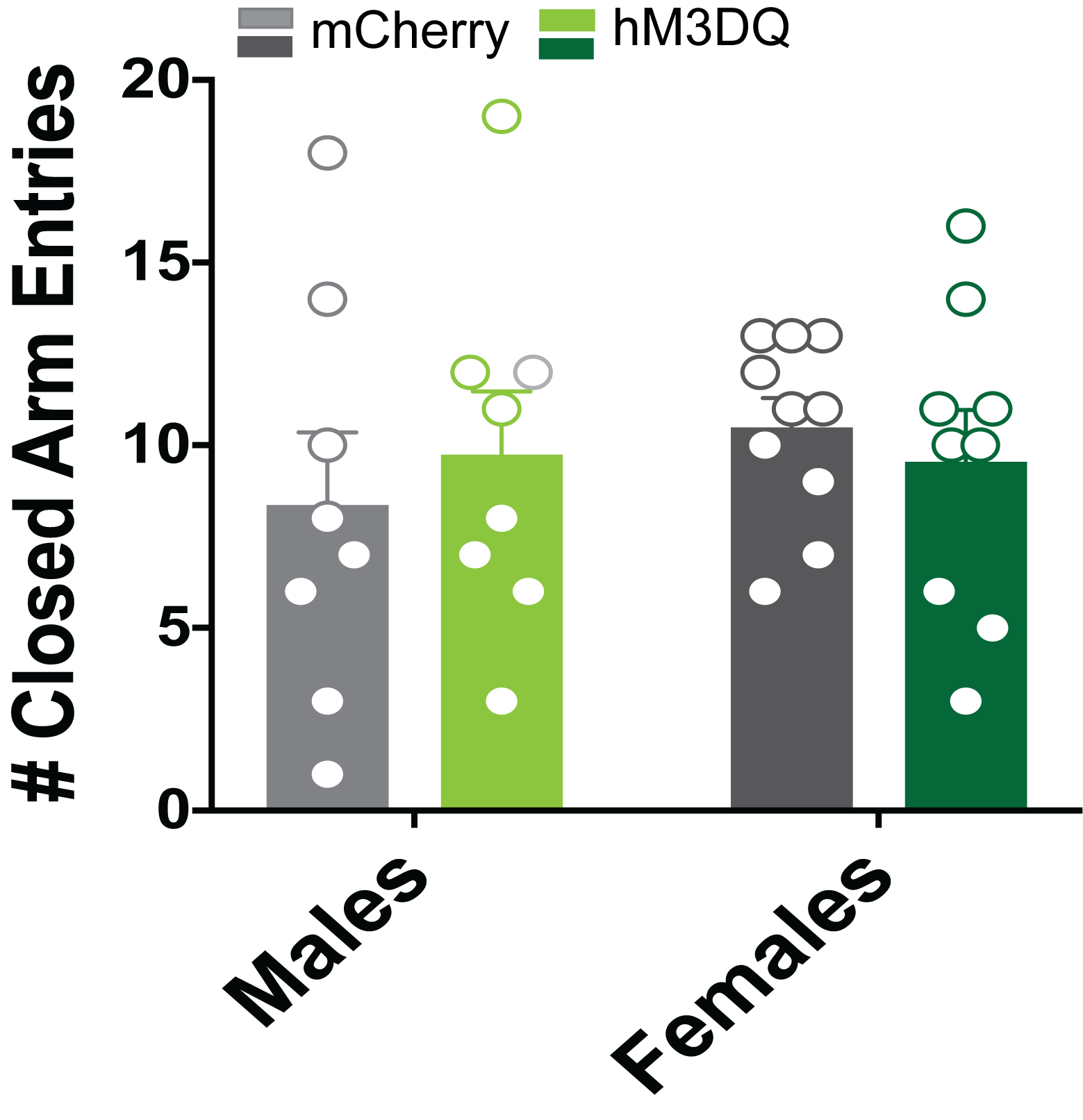

Supplement: Figure 7-1 — Closed arm entries were not altered on the EPM in mCherry-injected and hM3DQ-injected mice. The number of closed arm entries was not significantly different between mCherry-injected and hM3DQ-injected male and female mice. N = 8/group for males and N = 9–12/group for females. Download Figure 7-1, TIF file. [file enu-eN-MNT-0043-23-s10.tif]

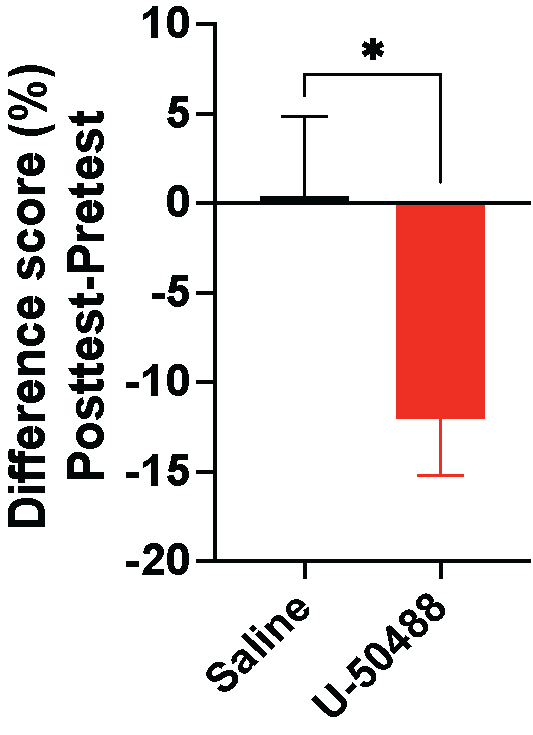

Supplement: Figure 8-1 — U-50488-induced CPA in C57BL/6J mice. Systemic injection of 2.5 mg/kg U-50488 produced CPA in WT mice. Difference score obtained by subtracting the percentage of time spent in drug-paired compartment before and after conditioning is shown for saline-injected and U-50488-injected mice. U-50488-injected mice showed CPA compared with mice that received saline injections on both sides of the chamber. *p = 0.032, t test, N =8–10 male mice/group. Download Figure 8-1, TIF file. [file enu-eN-MNT-0043-23-s11.tif]
