# Supplementary material for: A Cre Driver Line for Genetic Targeting of Kappa Opioid Receptor Expressing Cells
Source: eNeuro. 2023 Jul 11;10(7):ENEURO.0043-23.2023. doi: 10.1523/ENEURO.0043-23.2023 (PMC10348446; doi:10.1523/ENEURO.0043-23.2023)
Supplement: Table 3-1 — List of antibodies used with catalog numbers and dilutions. Download Table 3-1, DOCX file. [file enu-eN-MNT-0043-23-s02.docx]

| **Protein Target/ Antibody Name and Source** | **Primary Antibody Dilution** |
| --- | --- |
| P-ERK/ P-p44/42 MAPK (Mouse) (Cell Signaling Technology Danvers, MA #9106L) | 1:5000 |
| P-JNK/ P-SAPK/JNK (Rabbit) (Cell Signaling Technology Danvers, MA #4668S) | 1:5000 |
| P-p38/ P-p38 MAPK (Rabbit) (Cell Signaling Technology Danvers, MA #4511L) | 1:5000 |
| Total ERK (Rabbit) (Cell Signaling Technology Danvers, MA #9102L) | 1:5000 |
| Total JNK (Rabbit) (Cell Signaling Technology Danvers, MA #9258S) | 1:5000 |
| Total p38 (Rabbit) (Cell Signaling Technology Danvers, MA #9212L) | 1:5000 |

**Extended data Table 3-1:** List of antibodies used with catalog numbers and dilutions.
